# Aptamer-Based Fluorescence Detection and Selective Disinfection of Salmonella Typhimurium by Using Hollow Carbon Nitride Nanosphere

**DOI:** 10.3390/bios12040228

**Published:** 2022-04-09

**Authors:** Xinyi Liu, Jing Xu, Yang Lou, Chengsi Pan, Yin Zhang, Zhouping Wang

**Affiliations:** 1 State Key Laboratory of Food Science and Technology, Jiangnan University, Wuxi 214122, China; lwhyliuxinyi@163.com; 2 School of Food Science and Technology, Jiangnan University, Wuxi 214122, China; 3 International Joint Laboratory on Food Safety, Jiangnan University, Wuxi 214122, China; 4 Collaborative Innovation Center of Food Safety and Quality Control in Jiangsu Province, Jiangnan University, Wuxi 214122, China; 5 School of Chemical and Material Engineering, Jiangnan University, Wuxi 214122, China; yang.lou@jiangnan.edu.cn (Y.L.); cspan@jiangnan.edu.cn (C.P.); 6 Key Laboratory of Meat Processing of Sichuan, Chengdu University, Chengdu 610106, China; zhangyin@cdu.edu.cn; 7 National Engineering Research Center for Functional Food, Jiangnan University, Wuxi 214122, China

**Keywords:** hollow carbon nitride nanosphere, aptamer, *Salmonella typhimurium*, fluorescence detection, selective antibacterial

## Abstract

Hollow carbon nitride nanosphere (HCNS) was synthesized via the hard template method to improve the fluorescence characteristics, drug delivery ability, and photocatalytic activity. Blue fluorescent HCNS was utilized as a quenching agent and an internal reference to combine with Cy5-labelled aptamer (Cy5-Apt), resulting in an off-on fluorescence aptasensing method for the detection of Salmonella typhimurium (*S. typhimurium*). Under optimum conditions, this fluorescence assay presented a linear range from 30 to 3 × 10^4^ CFU mL^−1^ with a detection limit of 13 CFU mL^−1^. In addition, HCNS was also used as a drug carrier to load chloramphenicol (Cap) molecules. The Cap-loading amount of HCNS could reach 550 μg mg^−1^ within 24 h, whereas the corresponding Cap-release amount is 302.5 μg mg^−1^ under acidic and irradiation conditions. The integration of photocatalyst with antibiotic could endow HCNS-Cap with better disinfection performance. The bactericidal efficiency of HCNS-Cap (95.0%) against *S. typhimurium* within 12 h was better than those of HCNS (85.1%) and Cap (72.9%). In addition, selective disinfection of *S. typhimurium* was further realized by decorating aptamer. Within 4 h, almost all *S. Typhimurium* were inactivated by HCNS-Cap-Apt, whereas only 13.3% and 48.2% of *Staphylococcus aureus* and *Escherichia coli* cells were killed, respectively. Therefore, HCNS is a promising bio-platform for aptamer-based fluorescence detection and selective disinfection of *S. typhimurium*.

## 1. Introduction

Food products are susceptible to bacterial infections during processing, transportation, and storage, especially Campylobacter, *Salmonella*, *Listeria*, *Escherichia coli* (*E. coli*) O157:H7, *Staphylococcus aureus* (*S. aureus*), and *Bacillus cereus* [1]. Foodborne diseases caused by pathogenic bacteria pose a serious threat to the health of people and the development of the food industry [2]. Among these pathogens, *Salmonella typhimurium* (*S. typhimurium*) is one of the most dangerous Gram-negative bacteria, which can infect a series of hosts and cause a variety of diseases, including gastroenteritis and typhoid fever. As *S. typhimurium* widely exists in eggs, milk, and raw chicken products, it has raised continued concern in the food industry [3]. Therefore, it is particularly important to develop efficient methods for the detection and removal of *S. typhimurium*. 

Various approaches for detecting foodborne pathogens have been investigated, such as plate counting [4], enzyme-linked immunosorbent assays [5], surface-enhanced Raman scattering [6], microscopies [7], biosensors [8], and immune colloidal gold technique [9]. However, these detection methods are not ideal in specific application scenarios and functions [10]. Nanomaterial-based fluorescence detection has the advantages of high sensitivity, low detection limit, and fast response, which indicates a good prospect for the detection of pathogenic bacteria. Currently, graphitic carbon nitride (g-C_3_N_4_) is applied as a common fluorescent probe for food safety detection due to its high fluorescence quantum yield, good stability, satisfactory biocompatibility, and low toxicity [11]. In addition, aptamer as a molecular recognition module is also an important component of biosensors [12,13]. Aptamer-based sensors have received increasing attention because of their simple synthesis, ease of modification, high selectivity, and affinity [14]. Thus, fluorometric assays with excellent sensitivity and specificity could be constructed via the combination of g-C_3_N_4_ nanomaterials and dye-labelled aptamers according to mechanisms such as fluorescence resonance energy transfer (FRET) or photoinduced electron transfer (PET) [15,16]. For example, Liu et al. developed a ratiometric fluorescent aptasensor by using phenyl-doped g-C_3_N_4_ nanosheets as a quenching platform and 5-carboxy-x-rhodamine-labelled aptamer as a signal probe, which was applied to determine adenosine in serum samples with a detection limit of 6.86 μM [15].

Developing highly effective antimicrobial technology to remove pathogens is also essential to avoid food safety risks. As antibiotics can exhibit good antimicrobial efficacy even at low concentrations, they are widely used in the breeding industry for the prevention and treatment of bacterial diseases in animals [17]. Chloramphenicol (Cap) is a broad-spectrum antibiotic with superior antibacterial activity and strong inhibitory effect on both Gram-negative and Gram-positive bacteria [16]. The photocatalysis technology also reveals great potential in the field of antibacterials. Due to the semiconductor characteristic of photocatalysts, a variety of active species can be produced under light irradiation, such as h^+^, H_2_O_2_, •OH, •O_2_^−^. and ^1^O_2_, which could damage the cell membrane, cause the leakage of cellular content, and finally inactivate the bacteria [18]. The compound g-C_3_N_4_ is a popular metal-free organic photocatalyst, which has been studied frequently due to its visible light response, tunable chemical/electronic structure, and excellent photocatalytic activity [19]. In the past few years, g-C_3_N_4_ has also been employed as an antimicrobial agent for the removal of various bacteria. Thurston et al. demonstrated that the viability of Gram-negative *E. coli* O157:H7 and Gram-positive *S. aureus* was nearly completely inactivated by g-C_3_N_4_ film under visible light. Zhao et al. synthesized atomic single layer g-C_3_N_4_ nanosheets that could kill 2 × 10^7^ CFU mL^−1^ of *E. coli* completely under visible light within 4 h, whereas only about 3 log and 5 log of *E. coli* could be killed by bulk g-C_3_N_4_ and g-C_3_N_4_ nanosheets, respectively [20]. Although both antibiotic and photocatalysts are effective in killing pathogenic bacteria, the corresponding antibacterial rates are not yet satisfactory. Therefore, the combination of HCNS and Cap to achieve synergistic disinfection is a key factor in further improving the antibacterial rate and sterilizing efficiency.

For this purpose, a drug-loading strategy has been proposed that can utilize photocatalyst with nanostructure as a carrier to load antibiotics [21]. The drug release efficiency can be modulated by adjusting the pH or irradiation conditions. Due to its high surface and good spatial structure, g-C_3_N_4_ nanomaterials could be regarded as a promising carrier platform for drug-loading. Dong et al. prepared fluorescent poly (ethylene glycol)-g-C_3_N_4_ quantum dots (g-CNQDs-PEG) loaded with the anticancer drug adriamycin (DOX). The drug-loading system exhibited pH-sensitive release characteristics with a drug release rate up to 56% [22]. Liu et al. developed cyclodextrin-modified g-C_3_N_4_ nanosheets as a drug carrier, which were pH-responsive to release the loaded DOX at pH = 5 with a release rate of 80% [23]. As g-C_3_N_4_ nanomaterials can act not only as a photocatalytic antibacterial but also as a drug carrier, Cap molecules were loaded onto the structure of g-C_3_N_4_ for the construction of a controlled synergistic antibacterial system. Considering the high target affinity and specificity of Apt, HCNS-Apt would selectively enhance the inactivation. Song et al. developed TiO_2_ particles conjugated with ssDNA aptamer of *E. coli* for the disinfection of *E. coli* with high selectivity. Due to the interaction between target surface and aptamer, the proximity of functionalized TiO_2_ particles to *E. coli* induced rapid and efficient transfer of reactive oxygen species into bacteria cells [24]. Cheng et al. developed an aptamer-ampicillin conjugate for treating biofilms with a bifunctional coupling by linking the aptamer of bacterial flagella with ampicillin. The aptamer-ampicillin conjugate exhibited a unique antibacterial effect on the prevention and dissolution of biofilms [25]. Therefore, we further hypothesize the combination of aptamer as a specific recognition module with g-C_3_N_4_-based drug delivery system for the selective disinfection of *S. typhimurium*. 

In this work, hollow carbon nitride nanospheres (HCNS) were prepared by the hard template method to obtain improved fluorescence characteristics, drug-loading capacity, and photocatalytic activity. An off-on fluorescence aptasensing approach was developed by utilizing HCNS as a quenching agent and Cy5-labelled aptamer as a signal probe. The corresponding sensitivity and specificity for the detection of *S. typhimurium* were carefully investigated. In addition, a synergistic antibacterial system was constructed via the integration of Cap as an antimicrobial agent with HCNS as a drug carrier/photocatalyst. Moreover, selective disinfection function was further achieved by decorating aptamer as a recognition module on this HCNS-based drug delivery system. The corresponding antibacterial rate, sterilizing efficiency, and selectivity for the removal of *S. typhimurium* were evaluated.

## 2. Materials and Methods

### 2.1. Preparation of HCNS

HCNS was prepared by the hard template method [26,27]. A volume of 3.4 mL ammonia was dissolved in 100 mL ethanol solution (14.5 vol%) and stirred at 30 °C for 30 min, followed by the addition of 5.6 mL ethyl orthosilicate (TEOS) solution with vigorously stirring until the mixture became a uniform-color solution. After standing for 1 h, homogeneous non-porous silicon spheres were obtained. A mixture of 4.7 g TEOS and 1.8 g tetramethoxysilane (TMOS) was then added dropwise to the above solution to form a thin porous silicon shell around the dense silicon core. After remaining at ambient temperature for 3 h, the obtained product was centrifuged, dried at 70 °C, and heated at 550 °C for 6 h with a heating rate of 2 °C min^−1^. The calcined products were neutralized with 1 mol L^−1^ HCl solution and placed in a water bath at 60 °C for 4 h, resulting in the silica sphere template. Subsequently, the as-prepared template and cyanamide were added in a 100 mL round-bottom flask at the ratio of 1:5 and mixed by ultrasound under vacuum for 3 h. The resulting yellow solid mixture was calcinated at 550 °C for 4 h at a heating rate of 4 °C min^−1^. Finally, the calcined products were treated with 4 mol L^−1^ ammonium hydrogen fluoride solution for 12 h to remove the silica sphere template. After being centrifuged, washed with ultrapure water and ethanol, and dried, the final yellow product was HCNS. The bulk g-C_3_N_4_ (CN) synthesized from cyanamide via polycondensation at 520 °C for 4 h was selected as the reference g-C_3_N_4_ sample.

### 2.2. Fluorescence Aptasensing of *S. typhimurium*

For the determination of *S. typhimurium*, 100 μL HCNS suspension (62.5 μg mL^−1^), 10 μL (1 μM) Cy5-Apt (5′-Cy5-TAT GGC GGC GTC ACC CGA CGG GGA CTT GAC ATT ATG ACAG-3′-aptamer) solution, and 90 μL *S. typhimurium* solution with different cell densities were mixed for hybridization reaction and incubated at 37 °C for 30 min. The fluorescent spectra were then analyzed on a Hitachi F-7000 fluorescence spectrometer under the excitation wavelength of 640 nm and 380 nm for the fluorescence intensity measurements of Cy5 and HCNS, respectively. The relationship between the fluorescent enhancement (F-F_0_) of Cy5-Apt at 670 nm to the fluorescent intensity of HCNS at 450 nm (F_HCNS_) and the cell density of *S. typhimurium* solution was obtained. The linear range and detection limit of *S. typhimurium* for the HCNS-based fluorescent aptasensing assay were calculated by fitting the linear curve.

### 2.3. Real Sample Preparation and Measurements

Milk samples were purchased from supermarkets and pretreated in the biological cabinet. A 25 mL milk sample was placed into a conical flask containing 225 mL sterile saline. The real sample was prepared by adding 1 mL *S. typhimurium* solution with certain cell density to 9 mL of pretreated milk, which was used to compare the sensitivity between HCNS-based fluorescent aptasensing method and the plate counting method for the detection of *S. typhimurium*. For the plate counting method, 1 mL milk sample with certain cell density of *S. typhimurium* was placed into the aseptic dish, followed by the addition of 15 mL sterile LB solid medium. The dish was quickly rotated to thoroughly mix the culture medium and the bacteria solution. After being solidified and incubated at 37 °C for 24 h, the cell densities (in CFU mL^−1^) of the plates were estimated by the colony counting method. Each concentration group was carried out in triplicate.

### 2.4. Specificity Tests

To verify the specificity for *S*. *typhimurium*, the responses of HCNS-based fluorescent aptasensor towards some other bacteria, including *S. aureus*, *Bacillus stearothermophilus*, *Listeria monocytogenes*, *F’s dysentery, Bacillus*, and *Vibrio parahemolyticus*, were measured. The cell densities of all bacteria solutions were 3 × 10^5^ CFU mL^−1^. The specificity tests were performed using the same detection conditions as noted above.

### 2.5. Drug Loading and Release Experiments

The Cap molecules were loaded into HCNS via a simple dipping method. Separately, 30 mg Cap and 30 mg HCNS were dispersed into 50 mL ultrapure water under sonication for 30 min. The two solutions were then mixed by stirring under vacuum for 30 min and placed in a vacuum oven at 37 °C for 24 h. Subsequently, HCNS loaded with Cap (HCNS-Cap) was centrifuged, washed with ultrapure water, and dried at 60 °C. The concentration of the Cap solution was determined by high-performance liquid chromatography (HPLC) (Thermo fisher Ultimate3000). The drug-loading ratio (L) of HCNS was calculated by the equation L (wt%) = (m_0_−m_1_)/m_0_ (m_0_ represents the original amount of Cap before drug loading; m_1_ represents the remained amount of Cap after drug loading).

The drug release experiment was carried out by dispersing 25 mg HCNS-Cap into 25 mL phosphate buffer solution (PBS) at different pH (7.4, 6.5, 5.5) and stirring at 37 °C for 24 h. A 2 mL suspension was collected at regular intervals and then mixed with another 2 mL fresh PBS solution. The obtained supernatant was filtered with a 0.22 um membrane filter to determine the concentration of Cap solution by HPLC. The drug release ratio (R) of HCNS was calculated by the equation R (wt%) = m_2_/(m_0_−m_1_) (m_0_ represents the original amount of Cap before drug loading, m_1_ represents the remained amount of Cap after drug loading, m_2_ represents the released amount of Cap from HCNS-Cap).

### 2.6. Antibacterial Experiments

*S. typhimurium* was used to evaluate the synergistic antibacterial performance of HCNS-Cap. *S. typhimurium* was oscillated in LB liquid medium at 37 °C for 6 h, centrifuged, washed several times with sterile 0.9% NaCl solution, and then suspended in sterilized saline. The cell density of *S. typhimurium* was 3 × 10^5^ CFU mL^−1^. According to the actual drug-loading ratio and the drug-release ratio of HCNS-based drug delivery system, the dosage of HCNS, Cap, and HCNS-Cap was *cal*. 0.20, 0.06, and 0.31 mg mL^−1^, respectively. The antibacterial experiments were carried out at room temperature in a photochemical apparatus (XPA-7) equipped with a 500 W xenon lamp and a 420 nm cutoff filter. At certain time intervals, aliquots of suspension were sampled and serially diluted with sterile saline. A 0.1 mL diluent sample was spread on LB solid medium and cultured at 37 °C for 12 h. The densities of viable *S. typhimurium* cells (in CFU mL^−1^) were calculated by the plate colony counting method. The light control group tests were carried out under visible light without antibacterial agents, the dark control group tests were conducted in the dark without antibacterial agents, and the pH5.5 control group tests were performed under visible light or dark conditions without antibacterial agents. The pH value of PBS was 5.5 in HCNS, Cap, and HCNS-Cap groups. Each set of experiments was performed in triplicate. The glassware and saline solution were sterilized at 121 °C for 20 min.

### 2.7. Selective Disinfection Experiments

The aptamer of *S. typhimurium* was decorated on HCNS-Cap by adsorption. The 100 μL HCNS-Cap suspension (62.5 μg mL^−1^) and 10 μLCy5-Apt (1 μM) solution were incubated for 30 min. The antibacterial activity of HCNS-Cap-Apt was studied against different bacteria, including *S. typhimurium*, *S. aureus*, and *E. coli*. A volume of 18 μL bacteria solution (3 × 10^5^ CFU mL^−1^) was added to the 96-well plate, followed by the addition of 82 μL HCNS-Cap-Apt solution (0.2 mg mL^−1^). The antimicrobial count of each bacteria was performed in eight parallel groups. The light source was a 500 W xenon lamp equipped with a 400 nm cut-off filter. At certain time intervals, aliquots of suspension were sampled and diluted with sterile saline. A 100 μL diluent sample was spread on LB solid medium and cultured at 37 °C for 12 h. The viable bacteria cells densities (in CFU mL^−1^) were calculated by the plate colony counting method. 

## 3. Results and Discussion

### 3.1. Characterization of HCNS

**Figure 1:**
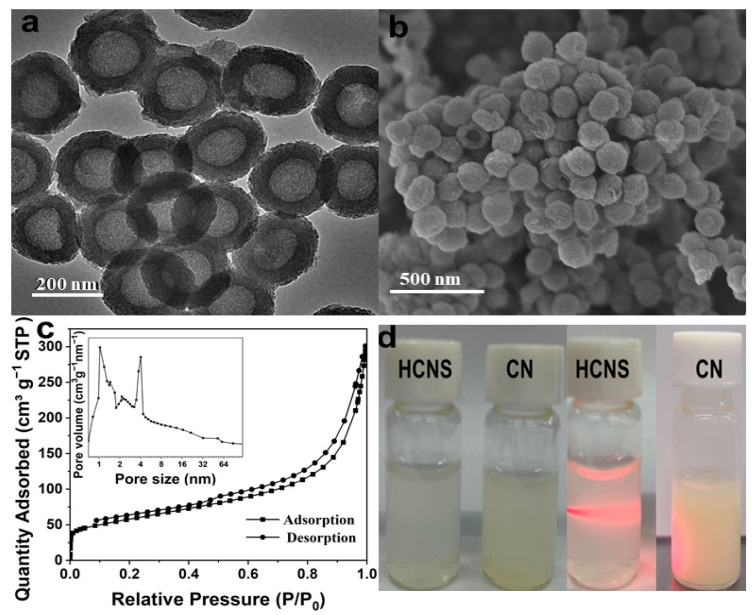
(**a**) TEM image and (**b**) SEM image of HCNS. (**c**) N_2_ adsorption-desorption isotherms of HCNS (inset: pore size distributions of HCNS). (**d**) Photographs of CN and HCNS dispersed in water (**left**), and the Tyndall effect of the HCNS dispersion (**right**).

The morphological structure was observed by transmission electron microscopy (TEM) and scanning electron microscopy (SEM). Compared to CN with a bulk structure at the micron level (Figure S1a,b in Supplementary Materials), HCNS presents a typical hollow spherical shape with a particle size of approximately 200 nm and a shell of approximately 50 nm (Figure 1a,b), demonstrating the smaller uniform morphology and larger internal space of HCNS. HCNS exhibits type-IV N_2_ adsorption-desorption isotherms with a high surface area (202.64 m^2^ g^−1^), a large pore volume (0.46 cm^3^ g^−1^), and a pore size in the range of 1–20 nm (Figure 1c), revealing the presence of abundant nanoporous channels in the internal space of HCNS, which are helpful to the mass transmission and charge migration in the reaction process. As shown in the X-ray diffractometer (XRD) patterns (Figure S2 in Supplementary Materials), the two characteristic peaks of HCNS at 12.9° and 27.4°, corresponding to in-plane repeated heptazine units and the planar stacking of conjugated C-N heterocycles, are weakened and widened in comparison with CN, indicating the shortened conjugate length of in-plane repeated structure and the reduced stacking of layered structure due to the transformation from the massive structure of CN into the hollow spherical structure of HCNS [28]. The results of XPS spectra (Figure S3 in Supplementary Materials) exhibit that the chemical state and composition of HCNS are inconsistent with CN, suggesting HCNS still retains the chemical structure of g-C_3_N_4_ after the morphology adjustment [29]. Compared with CN, the HCNS dispersion is more transparent and stable at 4 °C without the observation of aggregation (Figure 1d). The occurrence of the Tyndall phenomenon and higher zeta potential (24.7 mV) of the HCNS dispersion further prove its excellent dispersibility and stability in water (Figure S4 in Supplementary Materials). 

### 3.2. Fluorescence Aptasensing of *S. typhimurium* Based on HCNS

#### 3.2.1. Fluorescence Characteristics of HCNS

The fluorescence properties of HCNS are critical to its performance in fluorescence assay. The relative fluorescence quantum yield (QY) of HCNS was measured by comparison of quinine sulfate (54.6% at 330 nm excitation). According to the measured absorbance value and fluorescence intensity (Figure S5 in Supplementary Materials), the calculated relative QY of HCNS is 7.76%, which is much higher than that of CN (3.89%) (Table S1 in Supplementary Materials). The above results show that the hollow and porous structure is beneficial to the reflection/scattering of incident light within the nanospheres, resulting in the improved fluorescence characteristics of HCNS.

The stability of HCNS is also important to the accuracy of fluorescence determination. Figure S6a in Supplementary Materials shows that HCNS has high photobleaching resistance and still retains more than 90% of fluorescence intensity after 1 h of continuous illumination. Moreover, the fluorescence intensity of HCNS suspension at 450 nm has been negligibly changed after half a month of storage (Figure S6b in Supplementary Materials), which demonstrates the excellent stability of HCNS. In addition, after adding NaCl solutions with different concentrations, the fluorescence intensity of HCNS suspension still remain constant (Figure S6c in Supplementary Materials), indicating that HCNS has application potential in a high ionic strength environment. 

**Figure 2:**
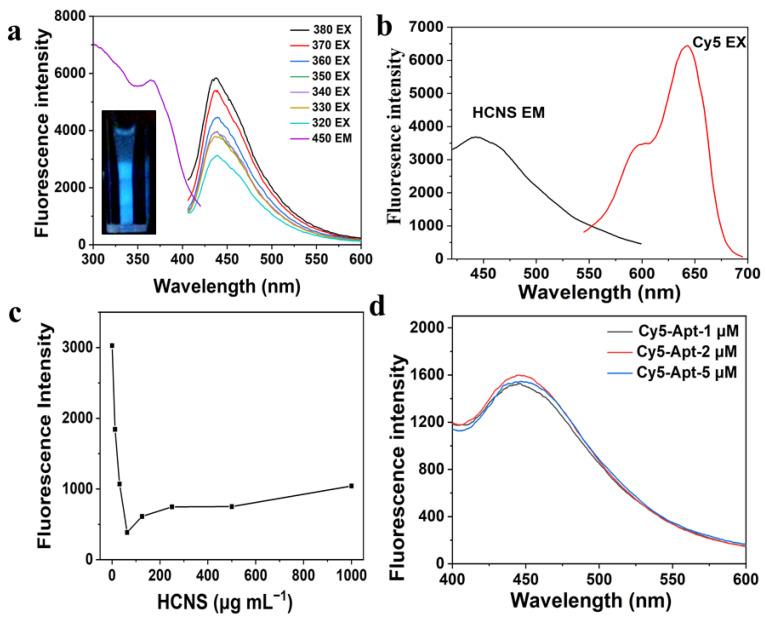
(**a**) The fluorescence excitation spectrum and the emission spectra of HCNS under different excitation wavelengths ranging from 330 to 380 nm (EX: excitation, EM: emission) (inset: the photograph of the HCNS suspension under 365 nm EX). (**b**) The emission spectra of HCNS and the excitation spectra of Cy5. (**c**) The fluorescence intensity of Cy5 with the addition of different concentrations of HCNS suspension. (**d**) The fluorescence intensity of HCNS with the addition of different concentrations of Cy5-Apt.

The emission spectrum of HCNS has one strong emission peak at 450 nm, which is consistent with the distinct blue luminescence of HCNS suspension under 365 nm UV light (Figure 2a). Furthermore, when the excitation wavelength varies from 330 nm to 380 nm, the emission peak stays at 450 nm, indicating the emission spectrum of HCNS is excitation-independent. Because the maximum emission intensity was realized at the excitation of 380 nm, it was selected as the excitation wavelength of HCNS. As shown in Figure 2b, as the emission spectra of HCNS excited at 380 nm did not overlap with the excitation spectra of Cy5 emitted at 670 nm, there was no FRET between HCNS and Cy5 [30]. Thus, the combination of HCNS and Cy5 can be applied to the construction of a fluorescence detection system based on the PET principle [15]. As shown in Figure 2c, under the excitation of 640 nm, the fluorescent intensity of Cy5 at 670 nm greatly decreased with the rising concentration of HCNS suspension, indicating that HCNS possesses strong fluorescent quenching ability toward Cy5. When the concentration of HCNS suspension was 62.5 μg mL^−1^, the maximum quenching rate of Cy5 reached approximately 91%. Furthermore, under the excitation of 380 nm, the fluorescence intensity of HCNS was not affected after the connection with various concentrations of Cy5-Apt, suggesting HCNS can be used as an internal reference for the subsequent construction of ratiometric fluorescence biosensor (Figure 2d).

**Scheme 1:**
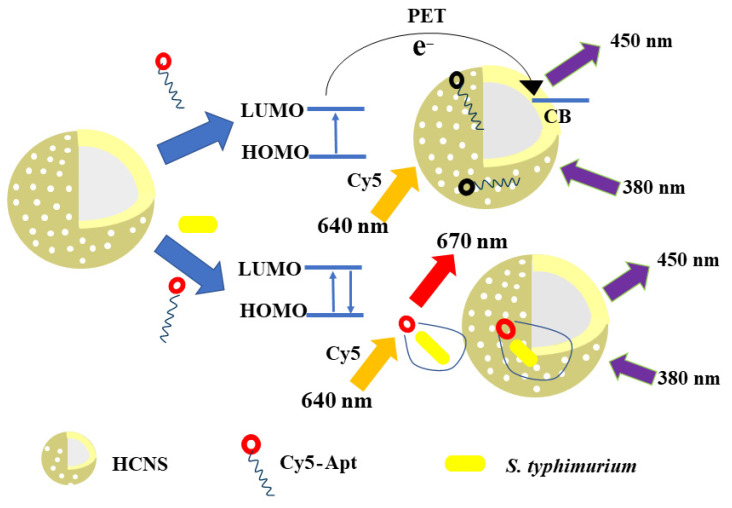
Schematic illustration of fluorescence aptasensing of *S. typhimurium* based on HCNS.

The off-on fluorescence aptasensor for the detection of *S. typhimurium* process is illustrated in Scheme 1. The maximal fluorescence excitation and emission wavelengths are 380 nm and 450 nm for HCNS and 640 nm and 670 nm for Cy5, respectively. In the absence of *S. typhimurium*, Cy5-Apt can be adsorbed on the surface of HCNS via π-π stacking and hydrophobic interactions between nucleobases and repeated heptazine planes [31]. Based on the PET principle, the fluorescence of Cy5 is effectively quenched by HCNS, whereas the fluorescence of HCNS is hardly changed. The fluorescence quenching is mainly attributed to the transfer of photogenerated electrons from the lowest unoccupied molecular orbital (LUMO) of Cy5 to the conduction band (CB) of HCNS [32]. In the presence of *S. typhimurium*, Cy5-Apt will preferentially bind to the target to form *S. typhimurium*-aptamer complex on account of the competition principle, resulting in the recovery of the fluorescence of Cy5 due to the vanishing of PET process. The ratio of the fluorescent enhancement of Cy5 to the inherent fluorescence of HCNS is used to quantitatively measure the cell density of *S. typhimurium*. Therefore, a ratiometric fluorescent aptasensing approach by utilizing HCNS as a quenching platform and an internal reference is constructed to realize the detection of *S. typhimurium* more accurately and reliably.

#### 3.2.2. Optimization of the Aptasensing Conditions

To achieve the sensitive detection of *S. typhimurium*, the effects of several experimental parameters including the concentration of HCNS suspension and the incubation time on the fluorescence intensity of Cy5 were carefully studied. The fluorescent enhancement (F-F_0_) of Cy5 after the addition of *S. typhimurium* was measured to choose the optimal condition (F_0_ and F represent the fluorescence intensity of Cy5 without and with *S. typhimurium*, respectively). As shown in Figure S7a (Supplementary Materials), the fluorescence enhancement achieves the maximum value at 62.5 μg mL^−1^ of HCNS suspension. Furthermore, the time-dependent fluorescence change of Cy5 after the addition of HCNS was monitored and the maximum fluorescence quenching took place in 30 min (Figure S7b in Supplementary Materials). The incubation time for the formation of *S. typhimurium*-aptamer complexes was also explored, and the fluorescent enhancement reached the top at 30 min (Figure S7c in Supplementary Materials). Therefore, 62.5 μg mL^−1^ of HCNS and 30 min of incubation time were selected for subsequent experiments.

#### 3.2.3. Fluorescence Determination of *S. typhimurium*

**Figure 3:**
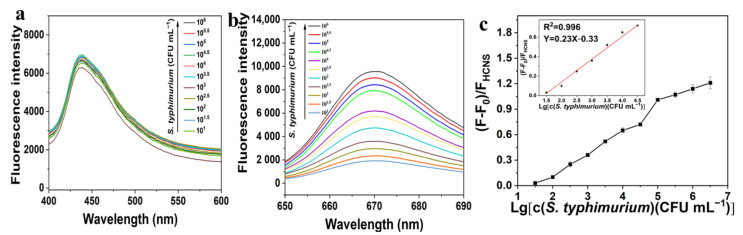
(**a**) Fluorescent emission spectra of HCNS (λ_ex_ = 380 nm) and (**b**) Cy5-Apt (λ_ex_ = 640 nm) after addition of different cell densities of *S. typhimurium*. (**c**) The ratio value of fluorescent intensity ((F-F_0_)/F_HCNS_) as a function of the cell density of *S. typhimurium*.

As shown in Figure 3a,b, with the increased cell density of *S. typhimurium* ranging from 0 to 3 × 10^6^ CFU mL^−1^, the fluorescence intensity of Cy5-Apt at 670 nm was gradually recovered, whereas the fluorescence intensity of HCNS at 450 nm was almost constant. The ratio of the fluorescent enhancement (F-F_0_) of Cy5-Apt at 670 nm to the fluorescent intensity of HCNS at 450 nm (F_HCNS_) increased with the rising cell density of *S. typhimurium* (Figure 3c). There is a good linear relationship between (F-F_0_)/F_HCNS_ and the cell density of *S. typhimurium* in the range of 30–3 × 10^4^ CFU mL^−1^, with a linear equation of (F-F_0_)/F_HCNS_ = 0.23 lg([*S. typhimurium*] (CFU mL^−1^) )− 0.33 (R^2^ = 0.996) and a limit of detection (LOD) of 13 CFU mL^−1^ (S/N = 3).

The applicability of our method was evaluated in milk samples. As presented in Table S2, the detected value of *S. typhimurium* is basically consistent with the added value. The obtained recovery varies from 96% to 116%, and the relative standard deviation (RSD) is in the range of 2–6%, indicating the feasibility of this HCNS-based fluorescence aptasensing method for the detection of *S. typhimurium* in real samples. 

**Figure 4:**
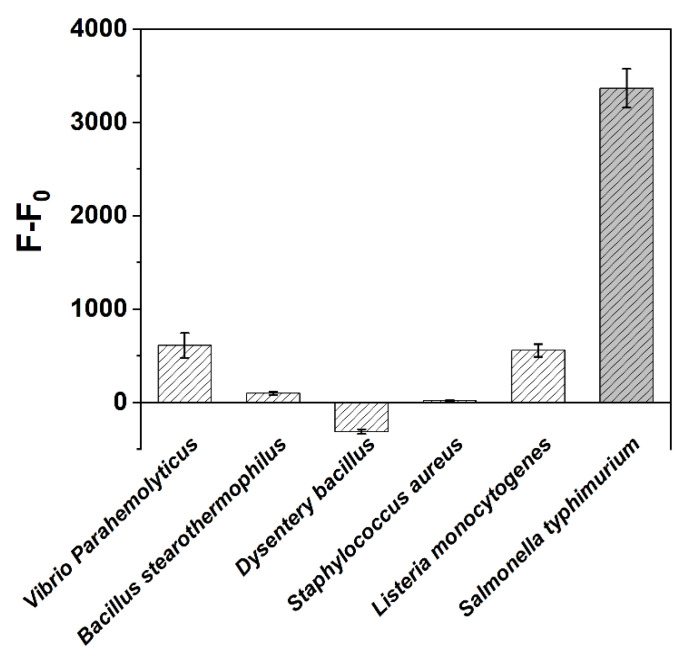
The fluorescence enhancement of Cy5 after the addition of 3 × 10^5^ CFU mL^−1^ of different bacteria (F_0_ and F represent the fluorescence intensity of Cy5 before and after adding different bacteria, respectively).

*S. aureus*, *Bacillus stearothermophilus*, *Listeria monocytogenes*, *Dysentery Bacillus,* and *Vibrio parahemolyticus* were selected to verify the specificity of this fluorescence assay for *S. typhimurium* determination. It is shown in Figure 4 that the fluorescence enhancement of Cy5 at 670 nm after the addition of *S. typhimurium* is significantly higher than that of the other five pathogenic bacteria. This is because aptamer would preferentially combine with *S. typhimurium* to form *S. typhimurium*-aptamer complex, resulting in the highest recovery degree of the fluorescence intensity of Cy5. Thus, the fluorescence aptasensing method based on HCNS has a good specificity towards *S. typhimurium*.

The detection performance of our method and other reported methods were compared and are listed in Table S3. Among all the methods, HCNS-based ratiometric fluorescence assay possesses the lowest LOD value, low time consumption, and acceptable linear range for *S. typhimurium* determination. Additionally, as the developed method has the advantages of high stability and simple operation, it shows a good application potential in the food industry.

### 3.3. Selective Disinfection of *S. typhimurium* by HCNS-Based Drug Delivery System Decorated with Aptamer

#### 3.3.1. Mechanism for the Selective Disinfection of *S. typhimurium*

**Scheme 2:**
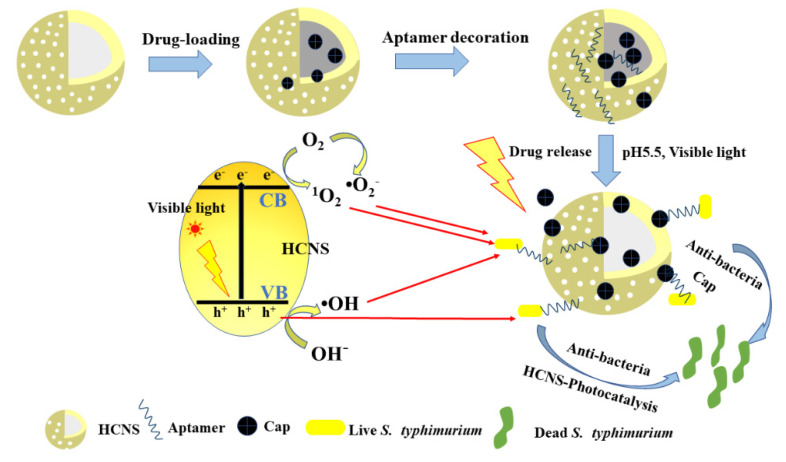
Schematic illustration of selective disinfection system by HCNS-based drug delivery system decorated with aptamer.

The mechanism of the selective disinfection of *S. typhimurium* by HCNS-based drug delivery system decorated with aptamer was proposed and is illustrated in Scheme 2. First, HCNS with large internal space and well-developed porosity is used as a drug carrier to load Cap molecules through electrostatic adsorption to form a drug-loading composite (HCNS-Cap). Second, aptamer is utilized as a recognition module and decorated on the surface of HCNS-Cap via π-π stacking and hydrophobic interactions, which could capture *S. typhimurium* cells specifically. Third, Cap molecules can be released slowly from HCNS by adjusting the pH and irradiation conditions so as to achieve the purpose of drug delivery. Finally, Cap molecules exhibit good antimicrobial efficacy on pathogenic bacteria, whereas HCNS can produce a certain amount of active species (h^+^, ^1^O_2_, •OH, •O_2_^-^) to attack bacteria under visible light irradiation [33]. The integration of photocatalyst with antibiotic can endow HCNS-Cap better disinfection performance towards *S. typhimurium.* Moreover, aptamers can attract *S. typhimurium* to the surrounding area of HCNS-Cap and shorten the distance between HCNS-Cap and bacteria, so it is feasible to realize the selective disinfection of *S. typhimurium*. In other words, compared to other bacteria, an HCNS-based drug delivery system decorated with aptamer (HCNS-Cap-Apt) could inactivate the *S. typhimurium* cells more preferentially. Therefore, a selective disinfection method by using HCNS as a drug carrier and photocatalytic antimicrobial agent is constructed to accomplish the removal of *S. typhimurium* more effectively and specifically [34].

#### 3.3.2. Drug Encapsulation and Release

**Figure 5:**
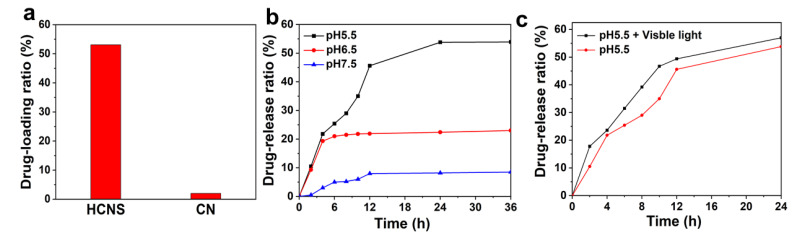
(**a**) The drug-loading rate of CN and HCNS. (**b**) The drug release rate of HCNS at different pH values. (**c**) The comparison of the drug release rate of HCNS at pH 5.5 with and without illumination.

Due to the high specific surface area and superior spatial structure, HCNS could be regarded as a promising carrier platform for drug-loading. In this study, Cap molecules with excellent antibacterial activity were selected to be drug-loaded in the structure of HCNS. As shown in Figure 5a, compared with CN, HCNS has a higher drug-loading rate due to its hollow nanosphere morphology. The drug-loading ratio of Cap reached 55.0% for HCNS and only 2.1% for CN. Explicitly, the Cap-loading amount of HCNS was 550.0 ug mg^−1^. The effect of pH value on the drug release rate was also compared in different PBSs (pH = 7.5, 6.5, 5.5) (Figure 5b). After 36 h of monitoring, it was found that the drug release rate of HCNS increased slowly and reached equilibrium at 12 h. The maximum Cap-release rate reached 53.0% at pH 5.5, which was much higher than at pH 6.5 (21.0%) and pH 7.5 (9.8%), indicating the acidic condition is more conducive to drug release. Moreover, the additional visible light irradiation could further improve the drug release rate at pH 5.5 to 55.0% (Figure 5c). Thus, under the condition of pH 5.5 and visible light, the maximum Cap-release amount of HCNS was 302.5 μg mg^−1^.

As shown in Figure S8 (Supplementary Materials), the average zeta potentials of HCNS and Cap solution are 25.0 mV and −2.5 mV, respectively. Thus, negatively charged Cap molecules can be successfully adsorbed on positively charged HCNS via electrostatic attraction, resulting in the decreasing zeta potential of HCNS-Cap to 22.5 mV. The differences in crystal and chemical structure of HCNS before and after Cap loading were analyzed by XRD, FTIR, and XPS measurements. As shown in Figure S9a,b (Supplementary Materials), the XRD pattern and FTIR spectrum of HCNS-Cap are almost similar to those of HCNS-Cap, indicating that the loaded Cap has not changed the crystal phase and chemical bonding of HCNS. The XPS survey spectra show that the surface elemental composition and percentage of HCNS-Cap are basically consistent with those of HCNS (Figure S10a and Table S4 in Supplementary Materials). The C 1*s* and N 1*s* spectra of HCNS-Cap are also in accordance with those of HCNS (Figure S10b,c in Supplementary Materials), suggesting the Cap molecules are mainly encapsulated in the hollow space and porous channels of HCNS and exert little influence on the surface chemical valence state of HCNS. As shown in the O 1*s* spectrum (Figure S10d in Supplementary Materials), the -OH peak of HCNS-Cap is enhanced in comparison with HCNS, which should be derived from the -OH groups of Cap molecules adsorbed on HCNS. As shown in Figure S11a,b (Supplementary Materials), the N_2_ adsorption-desorption isotherms and pore size distributions of HCNS and HCNS-Cap are quite similar, indicating that HCNS still retains the nanoporous structure after the loading of Cap. However, as listed in Table S5, the specific surface area, pore volume, and average pore width of HCNS-Cap (116.78 m^2^ g^−1^, 0.41 cm^3^ g^−1^, and 9.62 nm) are much lower than those of HCNS (202.64 m^2^ g^−1^, 0.46 cm^3^ g^−1^, and 12.89 nm), proving that the hollow space and porous channels of HCNS were occupied by Cap molecules during the loading process.

#### 3.3.3. Antibacterial Performance of HCNS-Based Drug Delivery System

**Figure 6:**
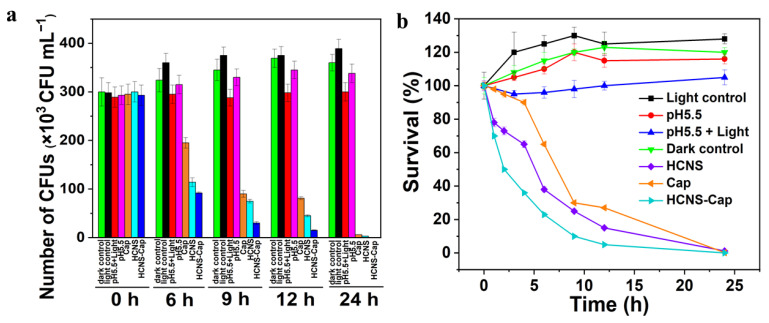
(**a**) The comparison of *S. typhimurium* colony number changes of HCNS, Cap, and HCNS-Cap during the disinfection process within 24 h. (**b**) The antibacterial performance of HCNS, Cap, and HCNS-Cap against *S. typhimurium*.

*S. typhimurium* cells were chosen as the target bacteria to evaluate the synergistic antibacterial performance of an HCNS-based drug delivery system. As shown in Figure 6a, the colony numbers of the dark control, the light control, the pH 5.5, and pH 5.5 + light groups did not decrease within 24 h, indicating that the weak acidic condition and visible light irradiation exhibit no antibacterial effect towards *S. typhimurium*. Under pH 5.5 and visible light conditions, in contrast, the colony numbers of *S. typhimurium* in HCNS and Cap groups were sharply reduced after 6 h, demonstrating the antibacterial effectiveness of photocatalyst and antibiotics. Moreover, compared with HCNS and Cap, the sterilization efficiency of HCNS-Cap was significantly improved. As shown in Figure 6b, after 6 h of disinfection, the antibacterial rate of HCNS-Cap on *S. typhimurium* reached 79.9%, whereas HCNS and Cap only killed 62.2% and 25.2% of bacteria cells, respectively. After 12 h, the bactericidal efficiency of HCNS-Cap was 95.0%, whereas 85.1% and 72.9% of bacteria were killed by HCNS and Cap groups, respectively. As the Cap-release amount of HCNS reached the maximum at 12 h, the bactericidal rate of HCNS-Cap was basically consistent with its drug release rate. The above results indicate that the combination of HCNS and Cap to achieve synergistic disinfection could further improve the antibacterial rate and sterilizing efficiency of *S. typhimurium.* Furthermore, the images of *S. typhimurium* colony on an agar plate in different groups during the disinfection process are compared in Figure S12 (Supplementary Materials), which can intuitively exhibit the synergistic antibacterial performance of HCNS-based drug delivery system.

**Figure 7:**
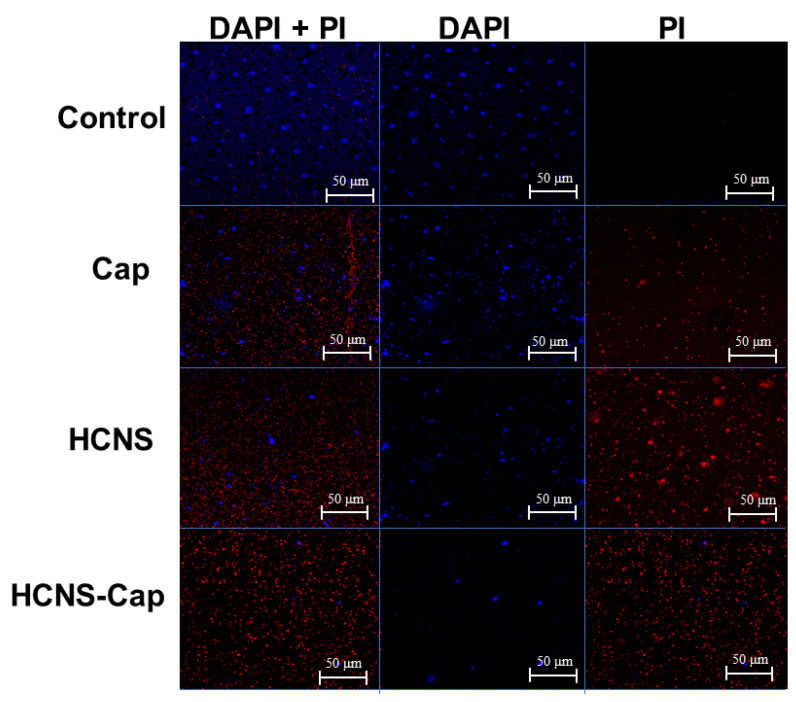
Fluorescent images of live and dead *S. typhimurium* cells treated by Cap, HCNS, and HCNS-Cap for 8 h.

To further verify the synergistic antibacterial activity of HCNS-Cap, fluorescent-based cell live/dead tests were performed by Polyimide (PI) and 4′,6-diamidino-2-phenylindole (DAPI) to stain the DNA of *S. typhimurium* cells. Red-fluorescent PI labels the dead bacteria, whereas blue-fluorescent DAPI labels the live bacteria. As shown in Figure 7, almost none of *S. typhimurium* cells were observed to be red fluorescent in the control group, indicating that few bacteria were killed. For HCNS and Cap groups, the bacterial cells displayed stronger red fluorescence, suggesting that more *S. typhimurium* cells were dead. In contrast, after being treated with HCNS-Cap, the bacterial cells exhibited the weakest blue fluorescence, indicating that the number of live *S. typhimurium* cells were minimal. Therefore, the integration of photocatalyst with antibiotics could endow an HCNS-based drug delivery system with excellent disinfection performance.

The reactive oxygen species (ROS) generated in the photocatalysis system of HCNS were studied by electron spin resonance (ESR) analysis. Hydroxyl radicals (•OH) and superoxide radicals (•O_2_^−^) were detected using DMPO as a spin probe in water and methanol, respectively, whereas singlet oxygen (^1^O_2_) was detected using TEMP as a spin probe in water. As shown in Figure S13, under visible light irradiation, HCNS exhibits a characteristic 1:2:2:1 quartet signal of •OH, a 1:1:1 triple signal of ^1^O_2_, and a weak signal of •O_2_^−^, suggesting that •OH, ^1^O_2_, and •O_2_^−^ are the main ROS participated in the photocatalytic antibacterial reaction of HCNS.

**Figure 8:**
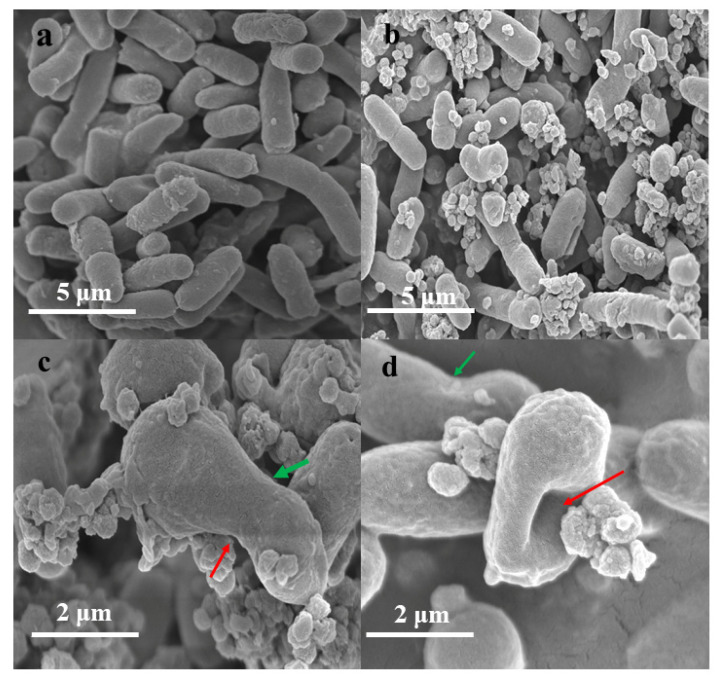
SEM images of (**a**) *S. typhimurium* cells alone, (**b**) *S. typhimurium* cells mixed with HCNS-Cap before disinfection and (**c**,**d**) after 4 h of disinfection. (The red arrow: the cell content has leaked; green arrow: the cell shape has changed).

Additionally, the morphological changes of *S. typhimurium* cells during the antibacterial process of HCNS-Cap were observed by SEM. As shown in Figure 8a, the initial *S. typhimurium* cells present a micrometer-sized rod shape with intact membranes. After being mixed with HCNS-Cap, *S. typhimurium* cells were tightly contacted with the nanospheres (Figure 8b), which is conducive to the antibacterial reaction. After 4 h of synergistic disinfection, the shape of *S. typhimurium* cells became irregular, with wrinkled cell membranes labeled by the green arrow and deformed cell walls labeled by the red arrow (Figure 8c,d). Thus, we speculate that *S. typhimurium* cells should be killed by the combination of the released Cap molecules and active species (h^+^, •OH, ^1^O_2_, •O_2_^−^) generated by HCNS under visible light [35]. A large number of active species with strong oxidation ability could attack the cell membranes, leading to the leakage of intracellular contents and the entry of Cap molecules and active species inside the cells, and finally facilitating the inactivation of *S. typhimurium*.

#### 3.3.4. Selective Disinfection of *S. typhimurium*

**Figure 9:**
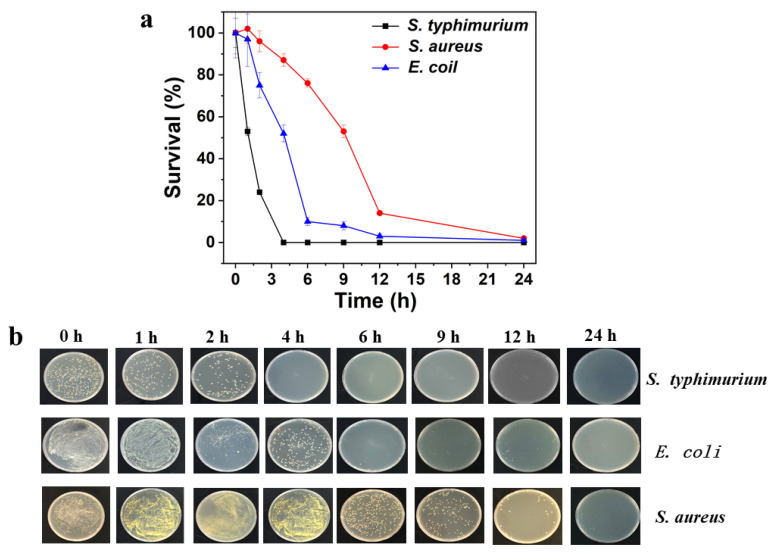
(**a**) The comparison of antibacterial activity of HCNS-Cap-Apt towards *S. typhimurium*, *E. coil*, and *S. aureus*. (**b**) The images of *S. typhimurium*, *E. coil,* and *S. aureus* colonies on agar plates during the disinfection process within 24 h treated by HCNS-Cap-Apt.

In addition, the aptamer is utilized as a recognition module and decorated on the surface of HCNS-Cap, which could capture *S. typhimurium* cells specifically. To verify the selective disinfection of *S. typhimurium* by HCNS-Cap-Apt, Gram-positive *S. aureus* and Gram-negative *E. coli* were selected as the reference bacteria. As shown in Figure 9, within 4 h, *S. typhimurium* cells were completely inactivated by HCNS-Cap-Apt. whereas only 13.3% of *S. aureus* cells and 48.2% of *E. coli* cells were killed, demonstrating the preferential inactivation of *S. typhimurium*. The above results should be mainly due to the specific binding between *S. typhimurium* and aptamer, which can attract *S. typhimurium* cells to the surrounding area of HCNS-Cap and shorten the distance between HCNS-Cap and *S. typhimurium*. As the attack on *S. typhimurium* cells becomes much easier, it is feasible to achieve the selective disinfection function of the HCNS-Cap-Apt system. 

## 4. Conclusions

In conclusion, multifunctional hollow carbon nitride nanospheres (HCNS) were synthesized via the hard template method. Due to the good fluorescent characteristics and high stability, HCNS was utilized as a quenching agent and an internal reference to combine with Cy5-Apt, resulting in a ratiometric fluorescent aptasensor for the detection of *S. typhimurium* with excellent sensitivity and specificity. Furthermore, HCNS was designed as a drug carrier to load Cap molecules on account of the large internal space and well-developed porosity. The integration of photocatalyst and antibiotics endowed HCNS-Cap with better antibacterial performance. In addition, selective disinfection of *S. typhimurium* was further realized by decorating aptamer as a recognition module on HCNS-Cap. Therefore, HCNS became an excellent platform for aptamer-based fluorescence detection and selective disinfection of *S. typhimurium*. This work provides a new idea to develop efficient approaches for the detection and removal of pathogenic bacteria and extends the application potential of g-C_3_N_4_ based nanomaterials in the field of food safety.

## Data Availability

Not applicable.

## Supplementary Materials

The following supporting information can be downloaded at: https://www.mdpi.com/article/10.3390/bios12040228/s1, Figure S1: TEM and SEM images of CN; Figure S2: XRD patterns of CN and HCNS Figure S3:XPS spectra of CN and HCNS. Figure S4: Zeta potentials of CN and HCNS. Figure S5: The fluorescence emission spectra under the excitation wavelength of 330 nm and (b) the UV-Vis absorption spectra of HCNS, CN and quinine sulfate. Figure S6: Photostability of HCNS. Figure S7: (a) The effect of the concentration of HCNS suspension on the fluorescent enhancement of Cy5. (b) Kinetic characteristic of the fluorescent intensity of Cy5 after the addition of 62.5 μg mL−1 of HCNS. (c) Kinetic characteristic of the fluorescent enhancement of Cy5 after the addition of 300 CFU mL-1 of *S. typhimurium*. Figure S8: Zeta potentials of HCNS, Cap and HCNS-Cap. Figure S9: (a) XRD patterns, (b) FTIR spectra of HCNS, Cap and HCNS-Cap. Figure S10: XPS spectra of HCNS and HCNS-Cap. Figure S11: (a) N_2_ adsorption-desorption isotherms and (b) pore size distributions of HCNS and HCNS-Cap. Figure S12: The images of *S. typhimurium* colony on agar plate. Figure S13: ESR spectra of HCNS under dark and visible light conditions. Table S1: Quantum yields of CN and HCNS in reference to quinine sulfate. Table S2: The recovery of *S. typhimurium* detection in milk samples. Table S3: The comparison of different methods for *S. typhimurium* determination.Table S4: The surface elemental content of HCNS and HCNS-Cap. Table S5. Specific surface area and pore volume of HCNS and HCNS-Cap.
