# Supplementary material for: Aptamer-Based Fluorescence Detection and Selective Disinfection of Salmonella Typhimurium by Using Hollow Carbon Nitride Nanosphere
Source: Biosensors (Basel). 2022 Apr 9;12(4):228. doi: 10.3390/bios12040228 (PMC9027708; doi:10.3390/bios12040228)
Supplement: Supplementary file 1 [file biosensors-12-00228-s001.zip › biosensors-1666074-supplementary.pdf]

Supporting Information

Aptamer-Based Fluorescence Detection and Selective Disinfection of Salmonella Typhimurium by Using Hollow Carbon Nitride Nanosphere

Xinyi Liu [1,2,3,4], Jing Xu [1,2,3,4,*], Yang Lou [5], Chengsi Pan [5], Yin Zhang [6], Zhouping Wang [1,2,3,4,6,7,*]

[1] State Key Laboratory of Food Science and Technology, Jiangnan University, Wuxi 214122, China; lwhyliuxinyi@163.com
[2] School of Food Science and Technology, Jiangnan University, Wuxi 214122, China
[3] International Joint Laboratory on Food Safety, Jiangnan University, Wuxi 214122, China
[4] Collaborative Innovation Center of Food Safety and Quality Control in Jiangsu Province, Jiangnan University, Wuxi 214122, China
[5] School of Chemical and Material Engineering, Jiangnan University, Wuxi 214122, China; yang.lou@jiangnan.edu.cn (Y.L.); cspan@jiangnan.edu.cn (C.P.)
[6] Key Laboratory of Meat Processing of Sichuan, Chengdu University, Chengdu 610106, China; zhangyin@cdu.edu.cn
[7] National Engineering Research Center for Functional Food, Jiangnan University, Wuxi 214122, China
* Correspondence: xujing823@jiangnan.edu.cn (J.X.), wangzp@jiangnan.edu.cn (Z.W.)

Table of contents

1. **Supplementary Experiments**

    1.1 Reagents

    1.2 Characterization

    1.3 Quantum Yield Determination

    1.4 Optimization of detection conditions

    1.5 The activation of strains

    1.6 SEM observation of bacteria

    1.7 Fluorescence microscopy observation of bacteria

2. **Supplementary Figures**

3. **Supplementary Tables**

1. Supplementary Experiments

1.1 Reagents. Hydrochloric acid (HCl), anhydrous methanol (CH_3OH), ammonia ($NH_3 \cdot H_2O$), ethyl orthosilicate ($(C_2H_5O)_4Si$, TEOS), tetramethoxysilane ($C_4H_{12}O_4Si$, TMOS), sodium hydroxide (NaOH), sodium dihydrogen phosphate (NaH2PO4), disodium hydrogen phosphate (Na_2HPO_4), sodium acetate (CH_3COONa), glacial acetic acid (CH_3COOH), potassium bromide (KBr), quinine sulfate ($(C_{20}H_{24}N_2O_2)_2 \cdot H_2SO_4 \cdot 2H_2O$), Polyimide ($C_{35}H_{28}N_2O_7$, PI), 4',6-diamidino-2-phenylindole ($C_{16}H_{15}N_5 \cdot 2HCl$, DAPI) were purchased from Sinopharm Chemical Reagent Co. Ltd. 5,5-dimethyl-1-pyrroline-n-oxide (DMPO) and 2,2,6,6-tetramethyl-4-piperidine (TEMP) were purchased from Sigma-Aldrich Co. LLC. HPLC-purified Cy5-labelled aptamer (5′-Cy5-TAT GGC GGC GTC ACC CGA CGG GGA CTT GAC ATT ATG ACAG-3′-aptamer (Cy5-Apt)) was synthesized by Shanghai Sangon Biotech.[1] The ultrapure water used in the experiments was obtained from the Direct-Q3 ultrapure water preparation machine. *Salmonella typhimurium* (*S. typhimurium*) (ATCC 14028), *Staphylococcus aureus* (*S. aureus*), *Bacillus stearothermophilus*, *Listeria monocytogenes*, *F's dysentery bacillus, Vibrio parahemolyticus* and *Escherichia coli* (*E. coli*) were purchased from the American Strain Conservation Center. Milk was purchased from RT-Mart (Wuxi, China). All the reagents used in this study were analytically pure and used without further purification.

1.2 Characterization. The morphology of samples was observed by transmission electron microscopy (TEM, JEM-2100) and scanning electron microscopy (SEM, FEI inspect F50). The phase structure of the sample was studied by Bruker D2 phaser X-ray diffractometer (XRD) (CuKα, 30 kV, 10 mA). Fourier transform infrared spectroscopy (FTIR) spectra were collected by Nicolet iS10 spectrometer. X-ray photoelectron spectroscopy (XPS) spectra were measured on ESCALAB 250Xi spectrometer and calibrated with C 1*s* binding energy at 284.8 eV. Zeta potential was obtained on a Zetasizer Nano ZS analyzer. The fluorescence spectra were determined by Hitachi F-7000 fluorescence spectrometer. The specific surface area and pore size distribution were measured from the nitrogen adsorption-desorption isotherms at 77 K (Quantachrome, QDS-MP-30) using the Brunauer-Emmett-Teller (BET) theory. Electron spin resonance (ESR) was measured by Bruker EMXplus paramagnetic resonance spectrometer to determine the active species generated by the samples. DMPO and TEMP were used as spin probes for hydroxyl radical (•OH), superoxide radicals ($\bullet O_2^-$) and singlet oxygen ($^1O_2$), respectively.

1.3 Quantum Yield Determination. The relative quantum yield (QY) was determined using the standard substance comparison method.[2] Under the same conditions, the QY of HCNS and CN were measured in reference to the fluorescence intensity integral of quinine sulfate with known quantum yield. The calculation

formula of QY is as follows:

$QY = QY_{ref} \times (\eta^2/\eta_{ref}^2) \times (I/I_{ref}) \times (A_{ref}/A)$

where QY_{ref} is the quantum yield of quinine sulfate; η and η_{ref} are the refractive index of the sample and reference solvents, respectively, which are usually quite close and both considered as 1.33; I and I_{ref} are the integrated fluorescence intensity of the sample and reference, respectively; A and A_{ref} are the absorbance of the sample and reference at the excitation wavelength, respectively. To minimize the reabsorption effect, the absorbence in the 10 mm cuvette should be below 0.1.

1.4 Optimization of detection conditions. HCNS-based fluorescence aptasensor (HCNS-Cy5-Apt) was prepared by the combination of Cy5-Apt and HCNS via adsorption. For the optimization of HCNS concentration, 100 μL HCNS suspension with different concentrations (1 mg mL^{-1}, 500 μg mL^{-1}, 250 μg mL^{-1}, 125 μg mL^{-1}, 62.5 μg mL^{-1}, 31 μg mL^{-1}, 25 μg mL^{-1}, 10 μg mL^{-1}) and 10 μL Cy5-Apt (1 μM) solution were added to a 96-well plate and incubated at 37 °C for 30 min. The relationship between the fluorescence intensity of Cy5-Apt and the concentration of HCNS was recorded by enzyme microplate reader (Synergy H1) with 100 μs delay time and 1 ms detection time. Afterward, the relationship between the fluorescence enhancement of Cy5-Apt after the addition of 300 CFU mL^{-1} *S. typhimurium* and the concentration of HCNS was also recorded. For the optimization of incubation time, 100 μL of 62.5 μg mL^{-1} HCNS suspension and 10 μL (1 μM) Cy5-Apt solution was added to the 96-well plate for hybridization reaction, and then incubated at 37 °C for different times (15 min, 30 min, 45 min, 60 min, 75 min). The relationship between the fluorescence intensity of Cy5-Apt and incubation time was recorded by enzyme micro-plate reader (Synergy H1). Meanwhile, the relationship between the fluorescence enhancement of Cy5-Apt after the addition of 300 CFU mL^{-1} *S. typhimurium* and incubation time was also recorded.

1.5 The activation of strains. Gram-positive bacteria (*S. aureu* and *Bacillus stearothermophilus*) and Gram-negative bacteria (*Listeria* monocytogenes, *F's dysentery bacillus*, *Vibrio parahemolyticus* and *S. typhimurium*) were inoculated in 100 mL sterile Luria Bertani (LB) nutrient solution and cultured at 37 °C. When the OD value at 600 nm reached 0.3, the bacterial cells were collected by centrifugation (4000 rpm, 4 °C, 5 min), washed several times with sterilized saline, and then stored in sterilized saline for further use. The OD values of the strains were measured by SpectraMax M5/M5e multifunctional microplate analyzer. The experimental apparatus and saline solution were sterilized at 121 °C for 20 min.

1.6 SEM observation of bacteria. The morphological changes of *S. typhimurium* cells were characterized by SEM before and after treatment of HCNS-Cap under visible light. The bacterial suspension was centrifuged at 4000 rpm for 5 min to remove the supernatant, and the precipitation was fixed with 2.5% glutaraldehyde

overnight, washed twice with sterile saline, dehydrated with 30%, 50%, 70%, 90% and 100% ethanol for 20 min, respectively, and finally freeze-dried. SEM images of the bacteria were taken by an FEI Inspect F50 field emission electron microscope.

1.7 Fluorescence microscopy observation of bacteria. Fluorescent-based cell live/dead tests were performed to clarify the integrity of bacterial cellular membranes. The bacteria solution after the above antibacterial treatment was centrifuged and washed with saline. Then the precipitates were stained with PI (50 μg mL^{-1}) and DAPI (50 μg mL^{-1}) for 20 min, respectively. After being washed three times with saline to remove the excess dye, the bacterial cells were imaged using a fluorescence microscope Nikon Ts2R-FL.

2. Supplementary Figures

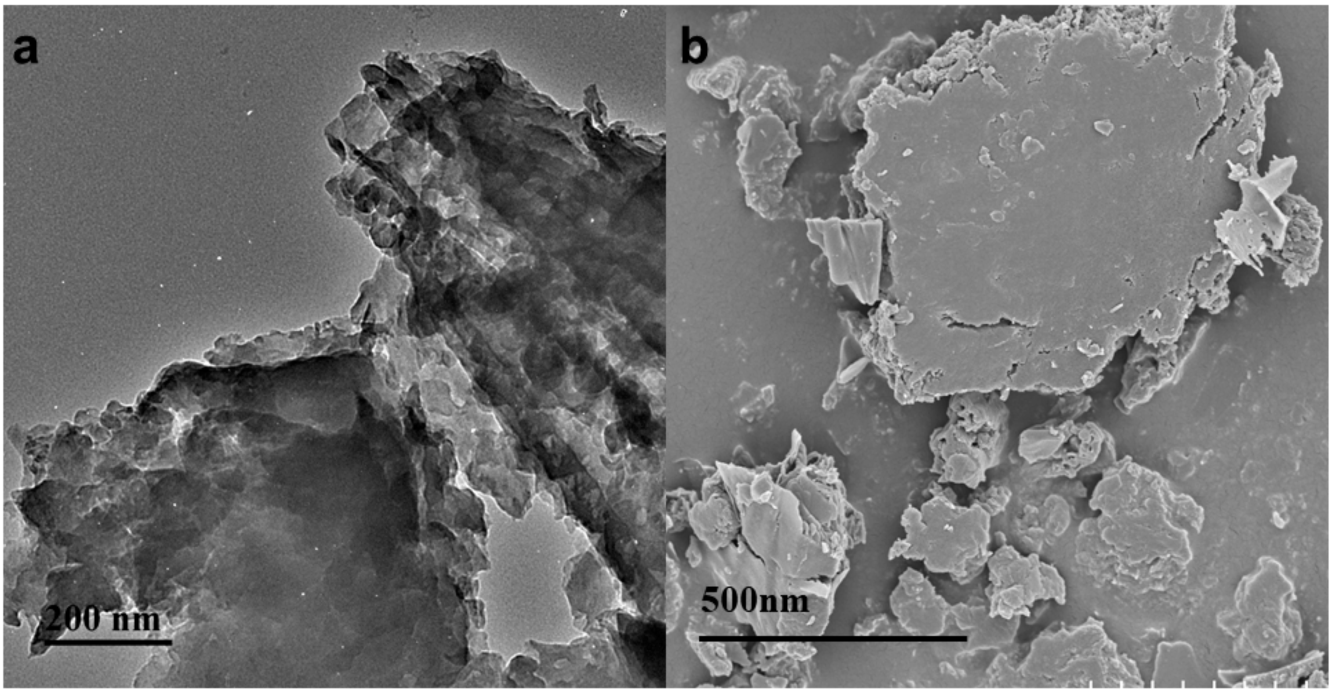

Figure S1. (a) TEM image and (b) SEM image of CN.

As shown in Figure S1a and S1b, CN presents a bulk structure at the micron level.

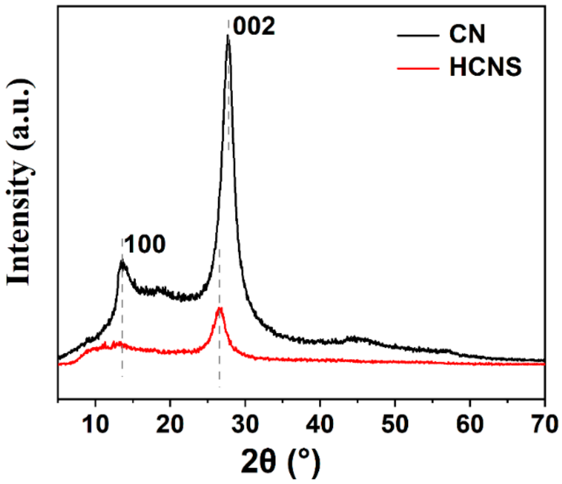

Figure S2. XRD patterns of CN and HCNS.

As shown in the X-ray diffractometer (XRD) patterns (Figure S2), the two characteristic peaks of HCNS at 12.9° and 27.4° corresponding to in-plane repeated heptazine units and the planar stacking of conjugated C-N heterocycles are weakened and widened in comparison with CN, indicating the shortened conjugate length of in-plane repeated structure and the reduced stacking of layered structure due to the transformation from the massive structure of CN into the hollow spherical structure of HCNS [3].

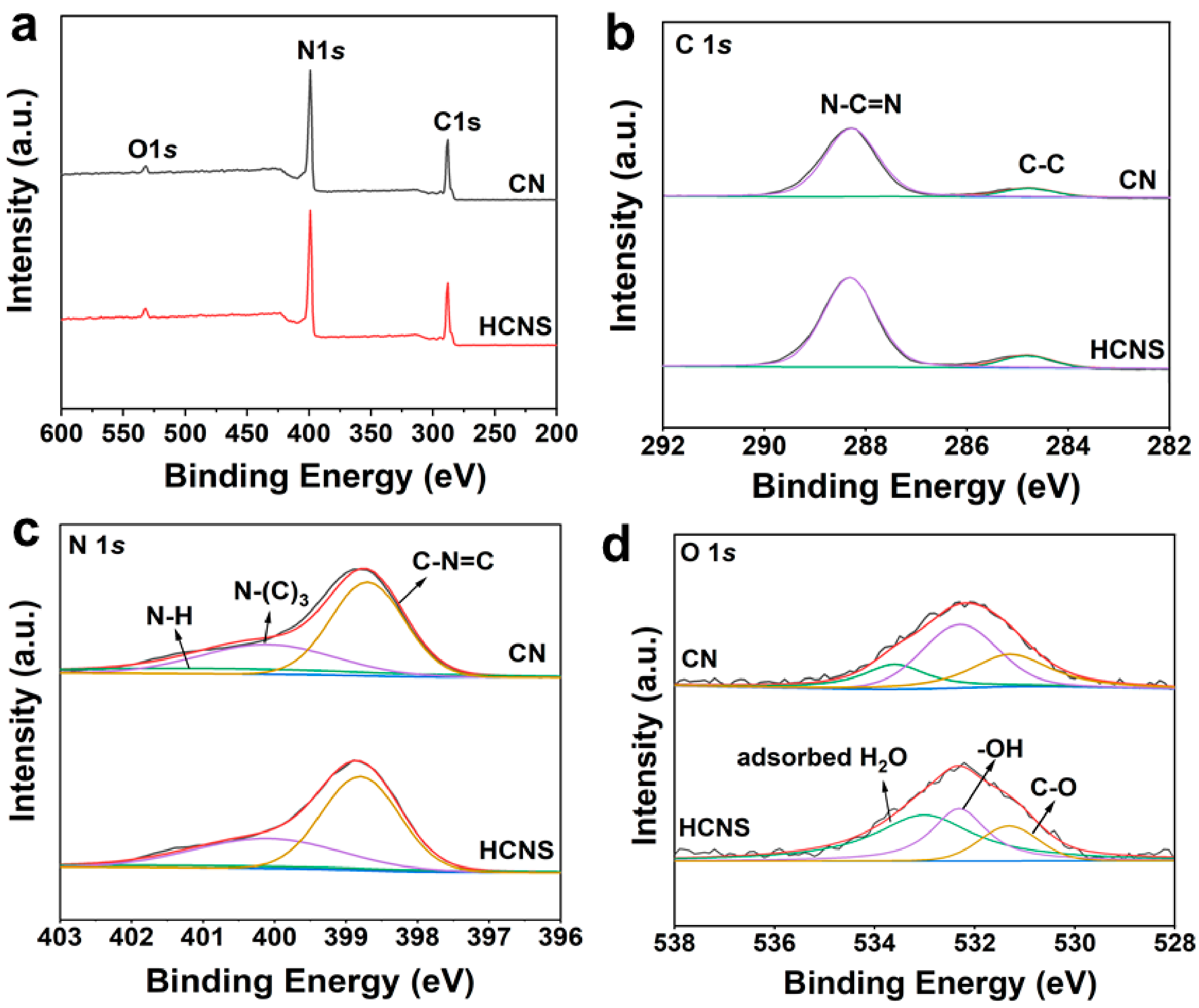

Figure S3. XPS spectra of CN and HCNS: (a) Survey, (b) C 1*s*, (c) N 1*s* and (d) O 1*s*.

As shown in the XPS survey spectrum (Figure S3a), HCNS mainly consists of C, N, and O elements. C 1*s* spectrum (Figure S3b) can be deconvolution into two peaks corresponding to the sp^2-hybridized N-C=N (287.8 eV) and sp^2 C-C bond (284.8 eV). N 1s spectrum (Figure S3c) can be fitted into three peaks at 398.6, 400.2, and 401.0 eV, arising from the sp^2-hybridized C-N=C, the N-$(C)_3$ and the-NH_x bonds involved in the tri-s-triazine rings, respectively.[4] O 1*s* spectrum (Figure S3d) shows three peaks at 533.4, 532.2 and 531.5 eV, attributing to the O in the adsorbed H_2O, the -OH bond, and the C-O bond. The XPS results exhibit that the chemical state and composition of HCNS are in consistent with CN.

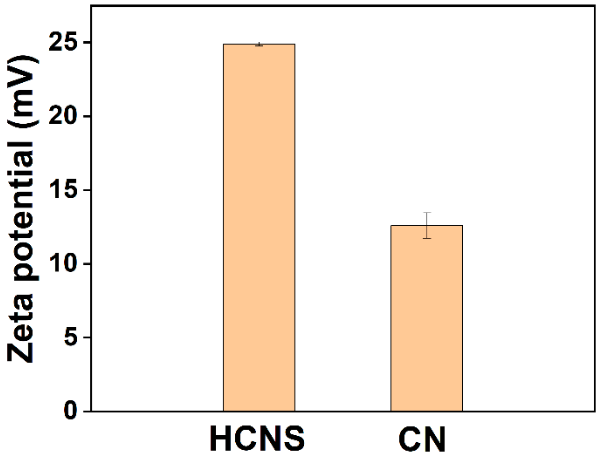

Figure S4. Zeta potentials of CN and HCNS.

The average Zeta potentials of HCNS and CN solution are 24.7 mV and 12.8 mV, respectively (Figure S4).

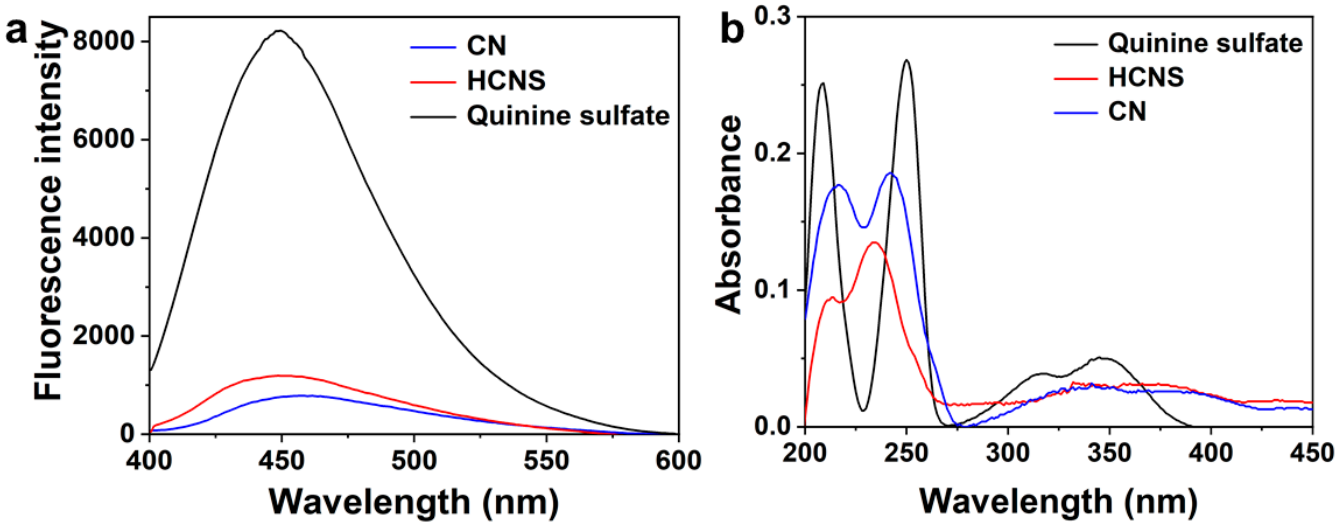

Figure S5. (**a**) The fluorescence emission spectra under the excitation wavelength of 330 nm and (**b**) the UV-Vis absorption spectra of HCNS, CN and quinine sulfate.

The fluorescence properties of HCNS are critical to its performance in fluorescence assay. The relative fluorescence quantum yield (QY) of HCNS was measured by comparison of quinine sulfate (54.6% at 330 nm excitation). According to the measured absorbance value and fluorescence intensity (Figure S5), the calculated relative QY of HCNS is 7.76%, which is much higher than that of CN (3.89%) (Table S1) The above results show that the hollow and porous structure is beneficial to the reflection/scattering of incident light within the nanospheres, resulting in the improved fluorescence characteristics of HCNS.

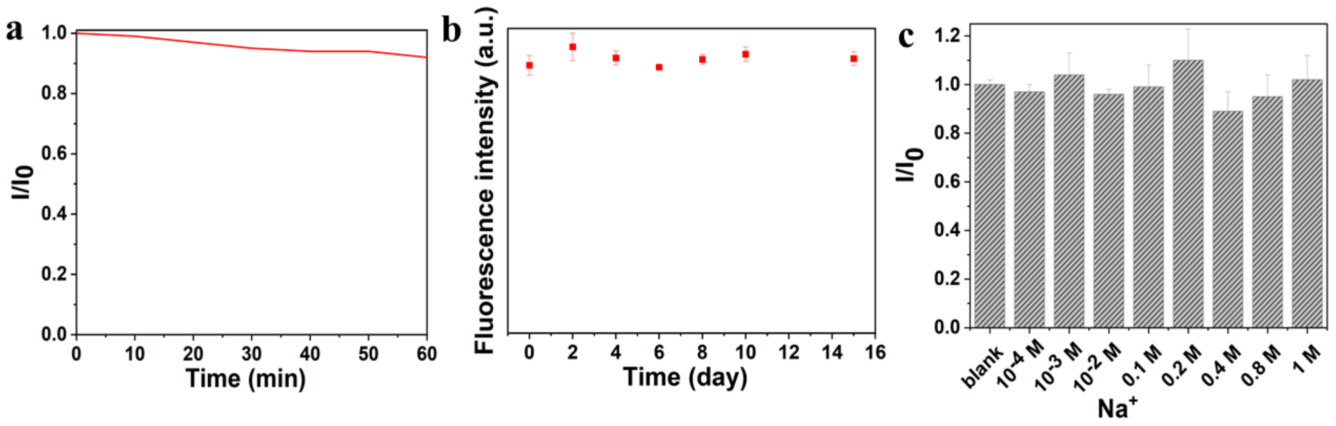

Figure S6. (**a**) Photostability of HCNS under irradiation (λ_{ex} = 380 nm) for 1 h (I_0 and I present the fluorescence intensity at initial and at certain moment, respectively). (**b**) The fluorescence intensity change of HCNS suspension stored for half a month. (**c**)The fluorescence intensity of HCNS in the presence of different concentrations of salt solutions (I_0 and I present the fluorescence intensity of HCNS before and after adding salt solutions, respectively).

The stability of HCNS is also important to the accuracy of fluorescence determination. Figure S6a shows that HCNS has high photobleaching resistance and still retains more than 90% of fluorescence intensity after 1 h of continuous illumination. Moreover, the fluorescence intensity of HCNS suspension at 450 nm has been negligibly changed after half a month of storage (Figure S6b), which demonstrates the excellent stability of HCNS. In addition, after adding NaCl solutions with different concentrations, the fluorescence intensity of HCNS suspension still remains constant (Figure S6c), indicating that HCNS has the application potential in high ionic strength environment.

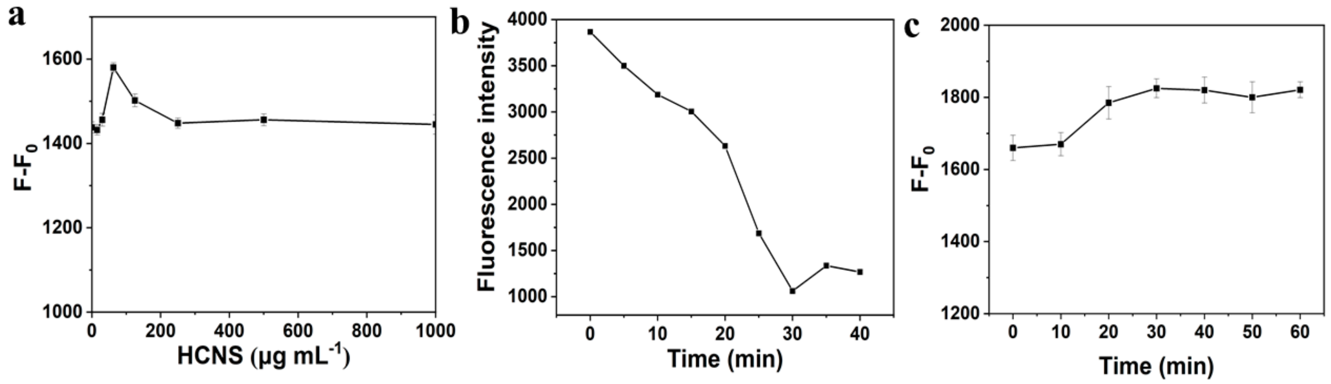

Figure S7. (**a**) The effect of the concentration of HCNS suspension on the fluorescent enhancement of Cy5. (**b**) Kinetic characteristic of the fluorescent intensity of Cy5 after the addition of 62.5 μg mL^{-1} of HCNS. (**c**) Kinetic characteristic of the fluorescent enhancement of Cy5 after the addition of 300 CFU mL^{-1} of *S. typhimurium*.

To achieve the sensitive detection of *S. typhimurium*, the effects of several experimental parameters including the concentration of HCNS suspension and the incubation time on the fluorescence intensity of Cy5 were carefully studied. The fluorescent enhancement ($F-F_0$) of Cy5 after addition of *S. typhimurium* was measured to choose the optimal condition (F_0 and F represent the fluorescence intensity of Cy5 without and with *S. typhimurium*, respectively). As shown in Figure S7a, the fluorescence enhancement achieves the maximum value at 62.5 μg mL^{-1} of HCNS suspension. Besides, the time-dependent fluorescence change of Cy5 after the addition of HCNS is monitored and the maximum fluorescence quenching takes place in 30 min (Figure S7b). The incubation time for the formation of *S. typhimurium*-aptamer complexes were also explored and the fluorescent enhancement reached the top at 30 min (Figure S7c). Therefore, 62.5 μg mL^{-1} of HCNS and 30 min of incubation time were selected for subsequent experiments.

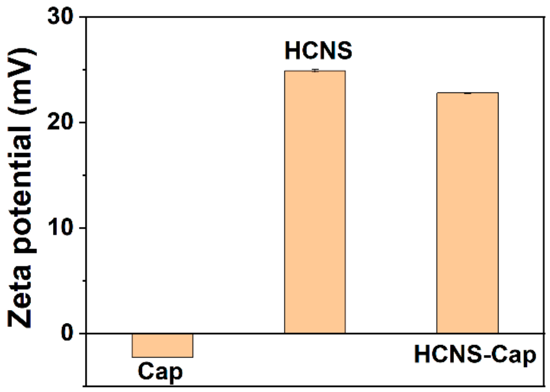

Figure S8. Zeta potentials of HCNS, Cap and HCNS-Cap.

As shown in Figure S8, the average Zeta potentials of HCNS and Cap solution are 25.0 mV and −2.5 mV, respectively. Thus, negatively charged Cap molecules can be successfully adsorbed on positively charged HCNS via electrostatic attraction, resulting in the zeta potential of HCNS-Cap decreases to 22.5 mV.[5]

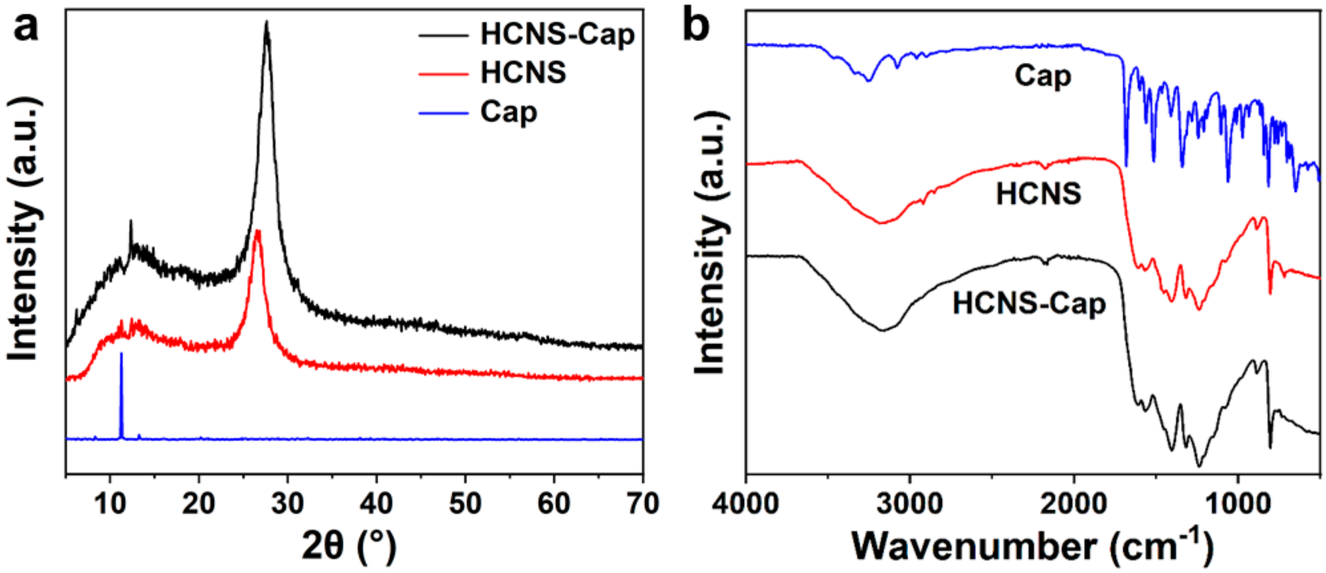

Figure S9. (**a**) XRD patterns, (**b**) FTIR spectra of HCNS, Cap and HCNS-Cap.

As shown in Figure S9a, the XRD pattern of HCNS-cap is almost similar to that of HCNS, indicating that the loaded Cap has not changed the crystal phase of HCNS. The chemical structure of Cap, HCNS and HCNS-Cap nanocomposites was studied by FTIR spectroscopy (Figure S9b). The spectrum of HCNS shows a wide peak at 2900–3500 cm^{-1}corresponded to the stretching vibration of O-H and N-H, a sharp peak at 809 cm^{-1} related to the bending vibrations of tri-s-triazine rings, and several strong bands at 1200–1650 cm^{-1} attributed to the typical stretching of CN heterocycles [6]. The characteristic peaks of HCNS-Cap are in accordance with that of HCNS, suggesting the loaded Cap also has no influence on the chemical structure of HCNS.

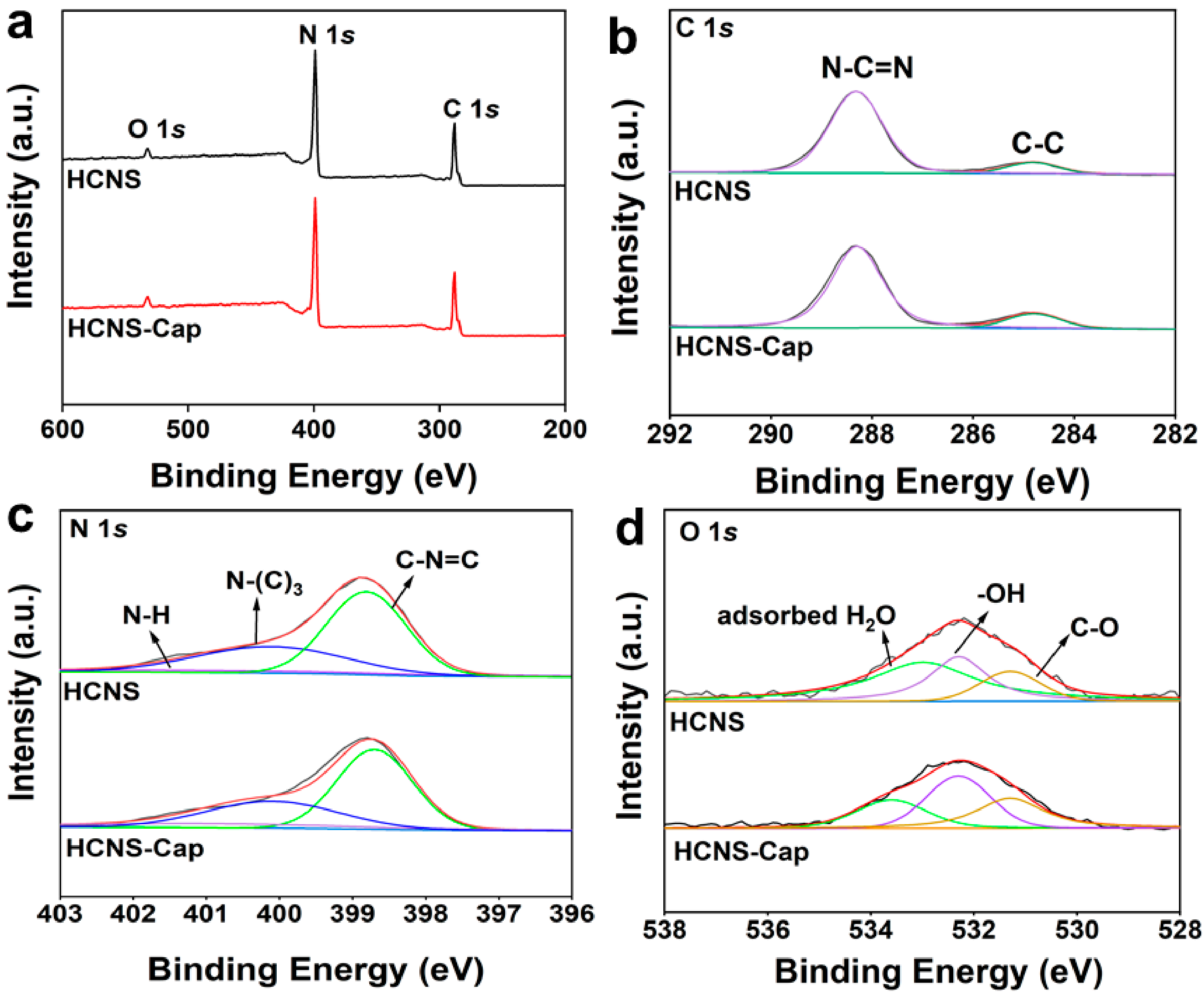

Figure S10. XPS spectra of HCNS and HCNS-Cap: (**a**) Survey, (**b**) C 1*s*, (**c**) N 1*s*, and (**d**) O 1*s*.

The XPS survey spectra show that the surface elemental composition and percentage of HCNS-Cap are basically consistent with those of HCNS (Figure S10a and Table S4). The C 1*s* and N 1*s* spectra of HCNS-Cap are also in accordance with those of HCNS (Figure S10b and S10c), suggesting the Cap molecules are mainly encapsulated in the hollow space and porous channels of HCNS and exert little influence on the surface chemical valence state of HCNS. As shown in the O 1*s* spectrum (Figure S10d), the -OH peak of HCNS-Cap is enhanced in comparison with HCNS, which should be derived from the -OH groups of Cap molecules adsorbed on HCNS.

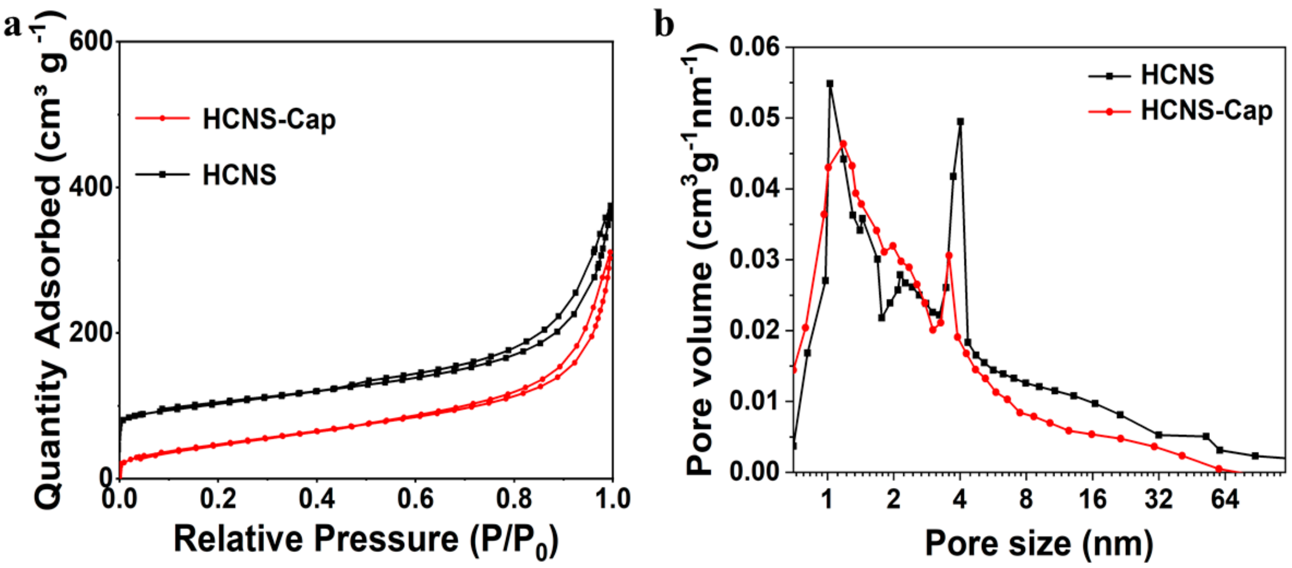

Figure S11. (**a**) N_2 adsorption-desorption isotherms and (**b**) pore size distributions of HCNS and HCNS-Cap.

As shown in Figure S11a and S11b, the N_2 adsorption-desorption isotherms and pore size distributions of HCNS and HCNS-Cap are quite similar, indicating that HCNS still remains the nanoporous structure after the loading of Cap.

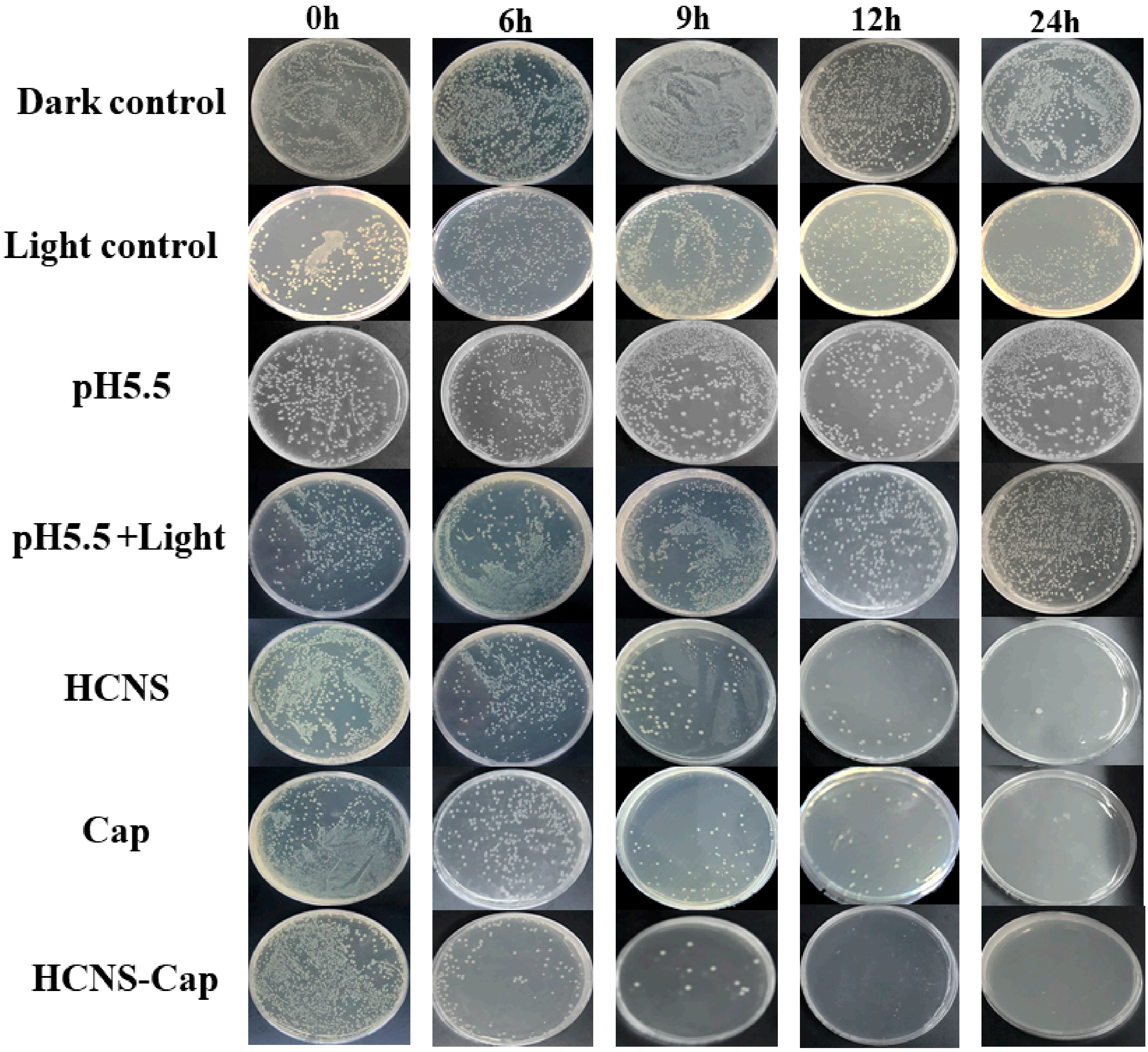

Figure S12. The images of *S. typhimurium* colony on agar plate during the disinfection process within 24 h treated by HCNS, Cap and HCNS-Cap.

The images of *S. typhimurium* colony on agar plate in different groups during the disinfection process are compared in Figure S12, which can intuitively exhibit the synergistic antibacterial performance of HCNS-based drug delivery system.

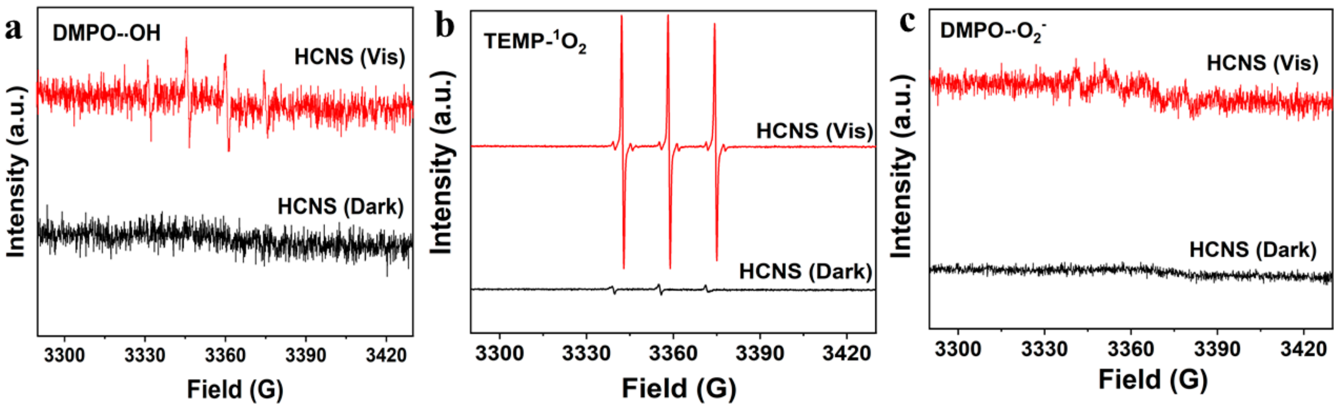

Figure S13. ESR spectra of HCNS under dark and visible light conditions for the detection of (a) •OH, (b) $^1O_2$ and (c) $•O_2^-$.

The reactive oxygen species (ROS) generated in the photocatalysis system of HCNS were studied by electron spin resonance (ESR) analysis. Hydroxyl radicals (•OH) and superoxide radicals ($•O_2^-$) were detected using DMPO as a spin probe in water and methanol, respectively, while singlet oxygen ($^1O_2$) was detected using TEMP as a spin probe in water [7]. As shown in Figure S13, under visible light irradiation, HCNS exhibits a characteristic 1:2:2:1 quartet signal of •OH, a 1:1:1 triple signal of $^1O_2$ and a weak signal of $•O_2^-$, suggesting that •OH, $^1O_2$ and $•O_2^-$ are the main ROS participated in the photocatalytic antibacterial reaction of HCNS.

3. Supplementary Tables

Table S1. Quantum yields of CN and HCNS in reference to quinine sulfate

Sample	I	$A_{330\ \mathrm{nm}}$	η	QY (%)
Quinine sulfate	662903.512	0.038	1.33	54.6
HCNS	108504.179	0.033	1.33	7.76
CN	66527.167	0.027	1.33	3.89

As listed in Table S1, the calculated relative fluorescence QY of HCNS is 7.76%, which is much higher than that of CN (3.89%).The above results show that the hollow and porous structure is beneficial to the reflection/scattering of incident light within the nanospheres, resulting in the improved fluorescence characteristics of HCNS.

Table S2. The recovery of *S. typhimurium* detection in milk samples.

Sample	Added ($CFU\ mL^{-1}$)	Plate counting ($CFU\ mL^{-1}$)	Our method ($CFU\ mL^{-1}$)	Recovery (%)	RSD (%)
1	30	36 ± 3	35 ± 2	116	5.7
2	3×10^3	3.29×10^3	2.89×10^3	96	2.7
3	3×10^6	3.47×10^6	2.96×10^6	98	3.6

The applicability of our method was evaluated in milk samples. As presented in Table S2, the detected value of *S. typhimurium* is basically consistent with the added value. The obtained recovery varies from 96% to 116%, and the relative standard deviation (RSD) is in the range of 2%−6%, indicating the feasibility of this HCNS-based fluorescence aptasensing method for the detection of *S. typhimurium* in real samples. Compared with the plate counting method, HCNS-based ratiometric fluorescence method is more accurate and less time consuming.

Table S3. The comparison of different methods for *S. typhimurium* determination

Methods	Time consumption (min)	Linear detection range (CFU mL^{-1})	Detection limit (CFU mL^{-1})	Reference
GO-based FRET method	35	10^3~10^8	100	[8]
Carbon-based fluorescence method	60	10^3~10^5	50	[9]
Loop-mediated isothermal amplification assay	60	10^3~10^7	34	[10]
SERS assay	1	10^2~10^7	27	[11]
GO-based time-resolved fluorescence method	45	10^2~10^6	25	[12]
HCNS-based ratiometric fluorescence method	30	30~3 × 10^4	13	This work

The detection performance of our method and other reported methods were compared and listed in Table S3. Among all the methods, HCNS-based ratiometric fluorescence assay possesses the lowest LOD value, less time consumption and acceptable linear range for *S. typhimurium* determination. Additionally, as our method has the advantages of high stability and simple operation, it shows a good application potential in food industry.

Table S4. The surface elemental content of HCNS and HCNS-Cap.

**Sample**	C (wt%)	N (wt%)	O (wt%)	H (wt%)
HCNS	46.64	45.48	5.67	2.21
HCNS-Cap	47.13	46.37	5.37	1.13

As listed in Table S4, the surface elemental percentage of HCNS-Cap are basically consistent with those of HCNS.

Table S5. Specific surface area and pore volume of HCNS and HCNS-Cap.

	Specific surface area (m^2 g^{-1})	Pore volume (cm^3 g^{-1})	Average pore width (nm)
HCNS	202.64	0.46	9.62
HCNS-Cap	116.78	0.41	12.89

As listed in Table S5, the specific surface area, pore volume and average pore width of HCNS-Cap (116.78 m^2 g^{-1}, 0.41 cm^3 g^{-1} and 9.62 nm) are much lower than those of HCNS (202.64 m^2 g^{-1}, 0.46 cm^3 g^{-1} and 12.89 nm), proving that the hollow space and porous channels of HCNS has been occupied by Cap molecules during the loading process.

REFERENCES

1. Raghavendra, J.; Harish, J.; Dwivedi, H.P.; Senthil Kumar, T.M.A.; Jaykus, L.A.; Schefers, J.; Srinand, S. Selection, characterization, and application of DNA aptamers for the capture and detection of Salmonella enterica serovars. *Molecular and Cellular Probes* **2009**, *23*, 20–28, doi:10.1016/j.mcp.2008.10.006.
2. Velapoldi, R.A.; Tonnesen, H.H. Corrected emission spectra and quantum yields for a series of fluorescent compounds in the visible spectral region. *J Fluoresc* **2004**, *14*, 465–472, doi:10.1023/B:JOFL.0000031828.96368.c1.
3. Wei, W.; Wei, Z.; Liu, D.; Zhu, Y. Enhanced visible-light photocatalysis via back-electron transfer from palladium quantum dots to perylene diimide. *Appl. Catal. B* **2018**, *230*, 49–57, doi:10.1016/j.apcatb.2018.02.032.
4. Xu, J.; Fujitsuka, M.; Kim, S.; Wang, Z.; Majima, T. Unprecedented effect of CO2 calcination atmosphere on photocatalytic H2 production activity from water using g-C_3N_4 synthesized from triazole polymerization. *Appl. Catal. B* **2019**, *241*, 141–148, doi:10.1016/j.apcatb.2018.09.023.
5. Lin, S.; Liu, X.; Tan, L.; Cui, Z.; Yang, X.; Yeung, K.W.K.; Pan, H.; Wu, S. Porous Iron-Carboxylate Metal-Organic Framework: A Novel Bioplatform with Sustained Antibacterial Efficacy and Nontoxicity. *ACS Appl. Mater. Interfaces* **2017**, *9*, 19248–19257, doi:10.1021/acsami.7b04810.
6. Liu, C.G.; Wu, X.T.; Li, X.F.; Zhang, X.G. Synthesis of graphene-like g-C3N4/Fe3O4 nanocomposites with high photocatalytic activity and applications in drug delivery. *RSC Adv.* **2014**, *4*, 62492–62498, doi:10.1039/c4ra10616d.
7. Xu, J.; Wang, Z.P.; Zhu, Y.F. Highly efficient visible photocatalytic disinfection and degradation performances of microtubular nanoporous g-C_3N_4 via hierarchical construction and defects engineering. *J Mater Sci Technol* **2020**, *49*, 133–143, doi:10.1016/j.jmst.2020.02.024.
8. Duan, Y.F.; Ning, Y.; Song, Y.; Deng, L. Fluorescent aptasensor for the determination of Salmonella typhimurium based on a graphene oxide platform. *Mikrochim Acta* **2014**, *181*, 647–653, doi:10.1007/s00604-014-1170-4.
9. Renjie, W.; Yi, X.; Tao, Z.; Yan, J. Rapid and sensitive detection of Salmonella Typhimurium using aptamer-conjugated carbon dots as fluorescence probe. *Anal. Methods* **2015**, *7*, 1701–1706, doi:10.1039/c4ay02880e.
10. Jin-Ho, K.; Cheoul, J.W. Detection of Salmonella Using the Loop Mediated Isothermal Amplification and Real-time PCR. *J. Korean Chem. Soc.* **2010**, *54*, 215–221.
11. Wang, P.X.; Sun, Y.; Li, X.; Wang, L.; Xu, Y.; He, L.L.; Li, G.L. Recent advances in dual recognition based surface enhanced Raman scattering for pathogenic bacteria detection: A review. *Anal. Chim. Acta* **2021**, *1157*, doi:10.1016/j.aca.2021.338279.
12. Xiaole, W.; Yukun, H.; Shijia, W.; Nuo, D.; Baocai, X.; Zhouping, W. Simultaneous detection of Staphylococcus aureus and Salmonella Typhimurium using multicolor time-resolved fluorescence nanoparticles as labels. *Int. J. Food Microbiol* **2016**, *237*, 172–179, doi:10.1016/j.ijfoodmicro.2016.08.028.
